# Health system costs and systemic treatment patterns by disease stage for 8577 people diagnosed with melanoma in New South Wales, Australia 2006–2019

**DOI:** 10.1371/journal.pone.0353408

**Published:** 2026-07-13

**Authors:** David E. Goldsbury, Anne E. Cust, Caroline Watts, Alison Pearce, Philip Haywood, Louisa G. Collins, Preston Ngo, Kirstie McLoughlin, Michael Caruana, Karen Canfell, Julia Steinberg, Marianne F. Weber

**Affiliations:** 1 The Daffodil Centre, The University of Sydney, and Cancer Council NSW, Sydney, Australia; 2 Sydney School of Public Health, The University of Sydney, Sydney, Australia; 3 Melanoma Institute Australia, The University of Sydney, Sydney, Australia; 4 Leeder Centre for Health Policy, Economics and Data, Sydney, Australia; 5 School of Public Health, University of Queensland, St Lucia, Queensland, Australia; 6 Queensland University of Technology, Brisbane, Australia; 7 Cancer Council Queensland, Brisbane, Australia; 8 Cancer Elimination Collaboration, Sydney School of Public Health, The University of Sydney, Sydney, Australia; Centro per lo Studio e la Prevenzione Oncologica, ITALY

## Abstract

**Background:**

Australia has the highest melanoma rates in the world. Melanoma is the third most common invasive cancer in Australia, and the number of *in situ* diagnoses is rapidly increasing. Treatment for non-localised stage melanoma is evolving, improving survival and increasing costs. We describe the costs and selected treatments by stage of disease for people diagnosed with melanoma in a large Australian cohort study.

**Methods:**

Questionnaire data for participants in the Australian 45 and Up Study (n = 267,357 recruited 2005–2009) were linked with cancer registrations, hospital records and Medicare claims records. To estimate the government healthcare costs attributable to melanoma, total costs for each participant with melanoma were compared to costs for matched cancer-free controls. Multivariable gamma regression was used to estimate factors associated with costs. We also examined the distribution of melanoma treatments by disease stage.

**Results:**

There were 8577 participants diagnosed with melanoma after recruitment (5026 *in situ* and 3551 invasive; median age 69 years at diagnosis). The mean excess per-person costs in the first year after diagnosis (‘initial phase’) ranged from $2794 Australian dollars (US$1825) for *in situ* melanoma to $70,070 for distant metastases. The corresponding mean annual costs in the continuing care phase were $1222 and $38,470, respectively. Of 195 participants with regional/metastatic melanoma diagnosed 2014–2019, 80 (41%) had a record of immunotherapy/targeted therapy and their unadjusted per-person costs were approximately $60,000 higher annually than the 115 participants without these treatments.

**Conclusions:**

Health system costs increased greatly with advancing melanoma stage, with higher costs linked to uptake of new immunotherapies/targeted therapies. Cost savings could arise from initiatives that detect and successfully treat melanomas at earlier stages, or prevent melanomas entirely.

## Introduction

Melanoma of the skin is the third most common invasive cancer in Australia, the country with the world’s highest incidence rate [1,2]. Although there are differences by age-group, the overall melanoma incidence rate in Australia has been increasing over time, with substantial implications for health service utilisation, planning, and health system costs. There were 16,878 people diagnosed with invasive melanoma and 27,585 with *in situ* melanoma (cancers located in the top layer of skin only) in Australia in 2021, up from 11,669 and 10,712, respectively, in 2011 [3]. Increases in melanoma incidence rates have also been observed in many other countries with predominantly fair-skinned populations, and may be due to increases in ultraviolet radiation exposure, increased awareness of and checks for skin cancer, and improvements in detection tools, while the ageing population also contributes to rising numbers of diagnoses [3–7].

Similarly, changes in available and recommended care can have implications for healthcare delivery, costs, and service utilisation. One such change is the increase in use of systemic therapies since around 2013, which have improved health outcomes but are high-cost, posing funding challenges for healthcare systems. In Australia, a potential future change in care could also come from a more structured approach to early detection, following the government announcement of $10 million (~US$6.5 million) in funding to develop a roadmap for a targeted skin cancer screening program [8].

The Australian health system includes a combination of a government-funded “universal” public system for permanent residents, supplemented by a private health system largely paid for by individuals, private health insurance premiums and government subsidies. Currently, recommended primary treatment of melanoma in Australia is driven by stage at diagnosis [9,10]. The recommended treatment for people with *in situ* or early-stage (stage I/II) invasive melanoma is complete surgical excision. People diagnosed with non-resectable stage III or stage IV melanoma, or those whose early-stage disease has progressed to regional or distant sites, are primarily treated with systemic therapies (immunotherapy and/or targeted therapy). Immunotherapy for melanoma was first listed on Australia’s Pharmaceutical Benefit Scheme for government subsidisation in 2013 [11] (Fig A in S1 File). In recent years, people with resectable stage III melanomas have started receiving neo-adjuvant (pre-surgical) immunotherapies [12] and new trials show benefit for this treatment for people with high-risk stage II disease [13].

Understanding the current health system costs of melanoma and their distribution is vital to project future healthcare resource utilisation and determine an optimal approach to reduce future cancer burden. There are different costing approaches, including model-based estimates and estimates using individual-level data. Previously, we used individual-level data to estimate the health system costs of cancer care in Australia for all cancer types combined, with summary costs for the 10 most common cancer types, including invasive melanomas, but without detail by stage [14]. A recent model-based study of melanoma and keratinocyte cancer costs in Australia reported detailed assumptions about cases, treatment pathways and micro-costing (specific costs for individual items/events) [15]. The study used decision-analytic models to estimate per-patient costs. We now provide a complementary individual-level data approach, using a distinct large-scale cohort study with a population-based sampling frame, to estimate costs of care by stage for people with melanoma, using a nested case-control design.

We aimed to estimate the health system costs of melanoma care in New South Wales (NSW), Australia’s most populous state, with detailed breakdowns by melanoma stage and phase of care. Our analysis also considered associations between individuals’ characteristics and melanoma treatment costs, and used the most recently available data to capture treatment patterns including the use of government-subsidised immunotherapy and targeted therapies in melanoma care.

## Materials and methods

### Source data

The Sax Institute’s 45 and Up Study included 267,357 residents of NSW, Australia, recruited in 2005–2009. Potential study participants were sampled from Services Australia’s Medicare enrolment database, which provides near-complete coverage of the Australian population. People aged >80 years and those in rural and remote areas were oversampled [16,17]. Participants completed a paper-based health and lifestyle questionnaire at recruitment (“baseline”) and gave written consent to have their questionnaire data linked with their health-related records held in routinely collected, administrative datasets. About 19% of those invited participated and participants included ~11% of the NSW population aged 45 years and over.

**Fig 1:**
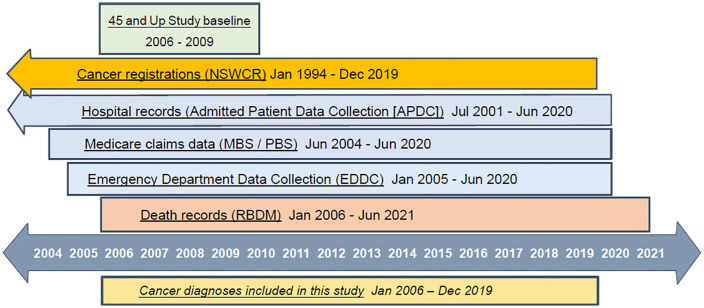
Data sources and date coverage. *MBS: Medicare Benefits Schedule; NSWCR: New South Wales Cancer Registry; PBS: Pharmaceutical Benefits Scheme; RBDM: Registry of Births, Deaths and Marriages. Death records also include cause of death information from the Cause of Death Unit Record File for Jan 2006 – Dec 2019*.

The linked health records spanned varying time periods, but all covered periods from the date of recruitment (or before) until at least 10 years after recruitment (Fig 1). This included reimbursements for government-subsidised prescription medicines in the Pharmaceutical Benefits Scheme (PBS), and outpatient and medical services and some in-hospital procedures subsidised through the Medicare Benefits Schedule (MBS), supplied by Services Australia. The Sax Institute linked these records using a unique identifier and deterministic matching. The Centre for Health Record Linkage [18] probabilistically linked other health records for study participants using data provided by the NSW Ministry of Health, including inpatient care data from all NSW hospitals in the Admitted Patient Data Collection (APDC), emergency care data in the Emergency Department Data Collection (EDDC), statutory cancer notifications in the NSW Cancer Registry (NSWCR; does not include keratinocyte/non-melanoma skin cancers), death notifications in the NSW Registry of Births, Deaths and Marriages, and cause of death data from the Australian Coordinating Registry’s Cause of Death Unit Record File. Approved study investigators had access to deidentified records only, in data extracts supplied by data custodians from 12 May 2022–21 December 2023 and accessed from then until manuscript submission.

### Study sample

**Fig 2:**
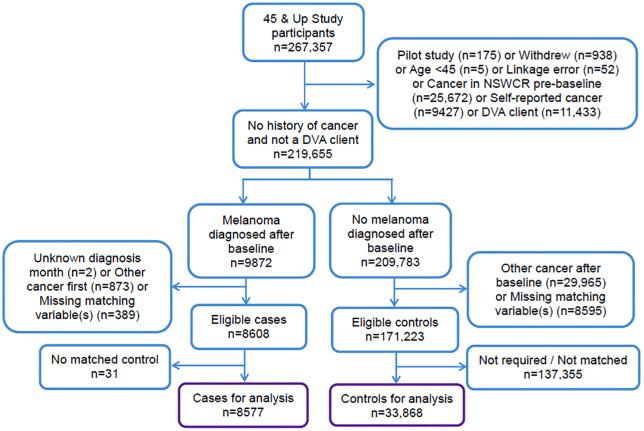
Study sample selection. *DVA: Department of Veterans’ Affairs; NSWCR: New South Wales Cancer Registry*.

From the 45 and Up Study baseline cohort we excluded participants (Fig 2): who were in the 2005 pilot, who later withdrew; with probable linkage errors; aged <45 years at baseline; with a NSWCR record of another invasive cancer prior to the incident melanoma; with a self-reported history of cancer (except keratinocyte cancers) if it was diagnosed before 1994 (earliest NSWCR date); with month of diagnosis unknown in NSWCR; or with melanoma first recorded on their death certificate. Those with healthcare subsidised by the Australian Government Department of Veterans’ Affairs (identified via self-report or any mention in healthcare records) were also excluded, as their prescription medicines are recorded in a different billing system not available for this study prior to 2013.

The study design was a nested case-control study, where ‘cases’ were 45 and Up Study participants with a NSWCR record of incident melanoma after baseline, up until December 2019. Melanoma diagnoses were identified using topography codes C43 (invasive) and D03 (*in situ*) from the International Statistical Classification of Diseases and Related Health Problems, Tenth Revision (ICD-10). Participants could have a single invasive melanoma recorded (data on diagnosis of multiple primary invasive melanomas were not available), and/or multiple *in situ* melanomas, so participants with melanoma were classified according to their first melanoma diagnosis. ‘Cases’ were matched to ‘controls’ who were participants with no NSWCR record of cancer at any time or self-reported cancer before 1994 (except keratinocyte cancers), and who were alive at the diagnosis date of their matched ‘case’. Up to four control participants were matched to each participant with melanoma by date of birth (±5 years), sex (female; male), Local Government Area of residence (153 areas in NSW) and smoking status (never; former, quit >15 years; former, quit ≤15 years; current) – all ascertained from baseline data. Participants with missing responses for any of the matching variables were excluded.

### Health system costs

Direct health system costs (healthcare system perspective) from the MBS and PBS data were based on the listed price in individual claim records. Inpatient hospital costs were derived from APDC records by linking the Australian Refined Diagnosis Related Group (AR-DRG) code for each hospitalisation to the relevant average admission cost recorded in the National Hospital Cost Data Collection (NHCDC) in the closest year available [19]. From July 2015 onwards, the dataset of private hospital records did not include AR-DRG codes, affecting approximately 30% of all admissions in this study. Using specialised software [20], we generated AR-DRG values for all APDC records, validated against the 70% of records with an AR-DRG in the original dataset. ED presentations were assigned average costs by triage category and discharge status, using estimates from the 2018/2019 NHCDC [19]. APDC and EDDC costs were combined to give hospital-based costs in selected analyses. Participants with no records in one of these datasets were assumed to not have used that healthcare during the study period. All cost values were converted to 2019 Australian dollars using the Australian Health Index [21] – we did not convert to a more recent year to avoid potentially introducing volatility caused by cost changes relating to the COVID-19 pandemic.

**Fig 3:**
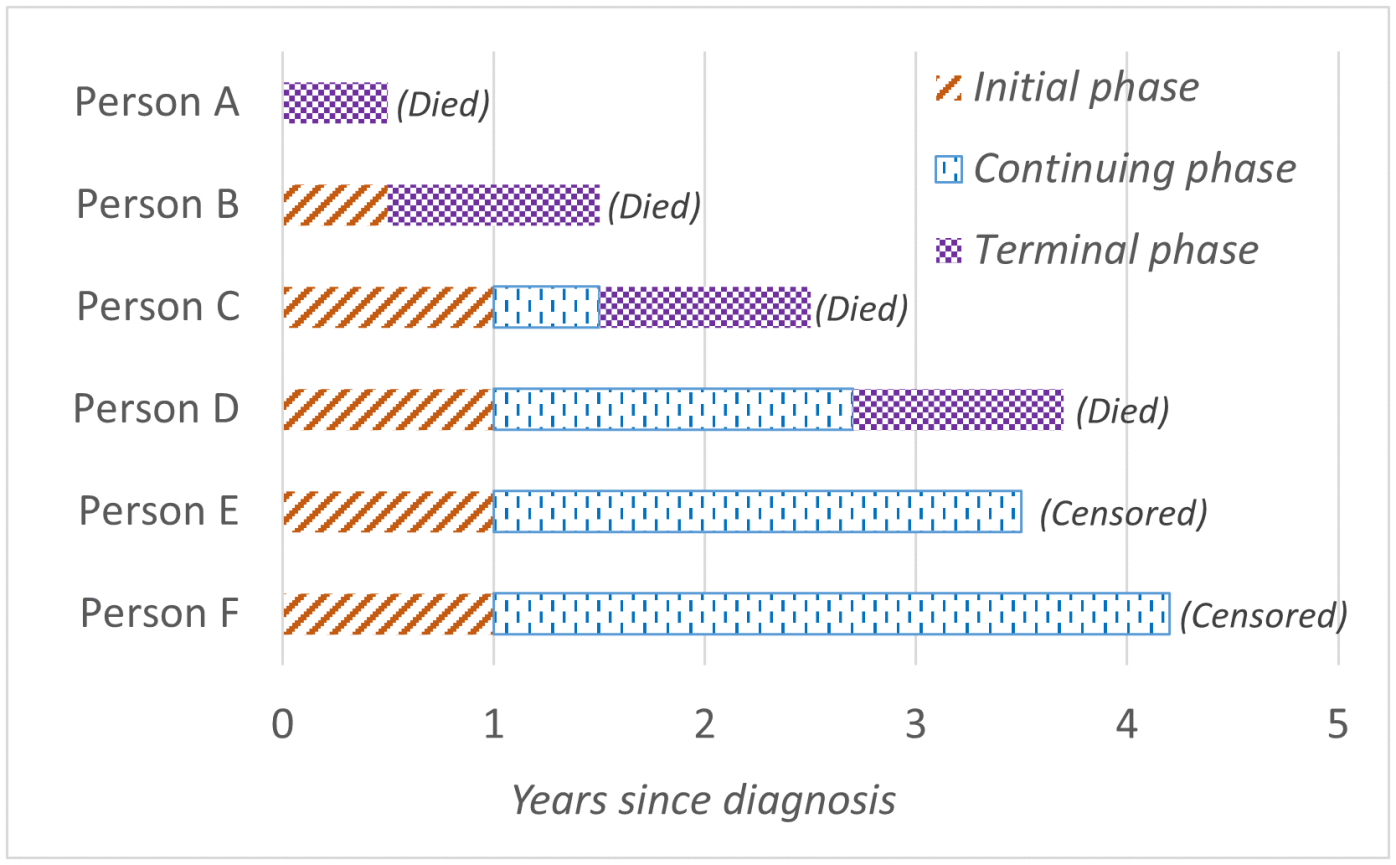
Hypothetical examples of follow-up time allocated to different phases of care, based on years since diagnosis and end of follow-up or death. *Initial phase: Up to 12 months from the date of diagnosis; Continuing phase: Time between initial phase and terminal phase or end of follow-up; Terminal phase: For people who died, the last 12 months of life. All are mutually exclusive*.

Costs were estimated for three phases of care: initial, continuing, and terminal (Fig 3). For participants with melanoma who died before July 2020, the final year up to death was designated the terminal phase. If they died ≤1 year after diagnosis, the terminal phase started at the diagnosis date and no costs were attributed to other phases. For participants with melanoma who survived >1 year but ≤2 years, the initial phase was the period from diagnosis until the start of the 12-month terminal phase. For participants with melanoma who survived at least two years, the first year after diagnosis was designated the initial phase, and the continuing phase was the period between the end of the initial phase and the start of the terminal phase or 30 June 2020. Costs for the continuing phase were annualised, and for this calculation we excluded case-control groups with a continuing phase of <3 months (<1% of groups).

We also estimated the costs for each 12-month period around a participant’s diagnosis date, from 2 years pre-diagnosis to 5 years post-diagnosis, along with monthly costs from 6 months pre-diagnosis to 6 months post-diagnosis. For participants with melanoma who died, we estimated the monthly end-of-life costs for the last 6 months relative to their death date.

### Treatment

A secondary aim was to describe melanoma-related treatment patterns, particularly the use of targeted therapies and immunotherapies, along with various other diagnostic and therapeutic procedures (Table A in S1 File). The PBS-listed targeted therapies during the study period were dabrafenib and/or tramatenib, vemurafenib and/or cobimetinib, and encorafenib and/or binimetinib. The immunotherapies were immune checkpoint inhibitors ipilimumab, pembrolizumab, and nivolumab. We estimated the proportion of participants who had each treatment, ascertained from APDC, MBS and PBS records from 30 days pre-diagnosis onwards. We also identified through MBS records the proportion of participants who had surgical treatment for any skin lesion, including keratinocyte carcinomas and benign lesions.

### Statistical analyses

The “excess costs” or “net costs” method aims to control for background healthcare utilisation, so that the difference in costs between the participant with cancer and their matched cancer-free control participants can be attributed to the condition of interest, without having to determine every healthcare resource specific to that condition [22]. The excess per-person costs due to melanoma were estimated by taking the total costs for a participant with melanoma and subtracting the mean costs of up to four matched control participants over the same time period. The excess costs are referred to here as “costs” unless otherwise specified. If the participant with melanoma was alive and any of their matched control participants died during the time period of interest, costs relative to the remaining matched control(s) were included in any subsequent calculations; we censored costs for participants with no remaining matched controls. Where relevant, costs were analysed using weights so that the (proxy) stage distribution in our study matched the distribution of all invasive melanomas diagnosed in NSW during 2006–2020 [23].

If the participant with melanoma died, then the included costs were censored at that date for annual and monthly calculations, and the case-control group was excluded from analyses for subsequent time periods. Means of costs were calculated, along with the proportions of costs contributed by each source (inpatient hospitalisations and ED presentations; prescription medicines in the PBS; services in the MBS). Cost medians, standard deviations, and inter-quartile ranges are also reported in the Supporting Information. Hospitalisations could span multiple time periods, so hospitalisation costs were apportioned across time periods using the proportion of days in each time period out of the total length of stay. Kaplan-Meier survival estimates were generated by disease stage.

Participants with melanoma were characterised by factors ascertained in multiple data sources (Table B in S1 File). We used NSWCR to ascertain: summary spread of disease at diagnosis (a proxy for “stage”: *in situ*; localised; regional; distant metastases; unknown), age at diagnosis, year of diagnosis, Breslow thickness of invasive melanoma at diagnosis (with <0.8mm as the reference category [24]), site of melanoma, and histology at diagnosis. We used the baseline questionnaire data for sex, body mass index (BMI), self-reported health, smoking status, country of birth, skin type, tannability of skin, remoteness of place of residence [25], quintile of socioeconomic disadvantage of place of residence [26], health insurance status, and highest education level. We identified comorbidities using hospital admissions in the 5 years prior to diagnosis, based on the 2011 modification of the Charlson Comorbidity Index (Table C in S1 File) [27].

We estimated costs with melanoma stage as the study factor. We also tested the association between costs in each phase of care and all characteristics described above using a multivariable gamma regression with a log link, simultaneously adjusting for all other covariates, and excluding participants with a missing value for any of the included covariates (~5% in each model). Gamma regression with a log link is a widely accepted method for modelling for health care costs, as it appropriately accounts for the right-skewed distribution of continuous cost values, including a long tail driven by a small proportion of patients with very high expenditure [28]. Any outlying values (standardised Pearson residual < −4 or >5) were excluded. Due to potential for negative excess costs for some people with cancer, in the regression analysis all cost estimates were “offset” by adding a set amount (+$50,000), so that all cost values were > $0 and could be included in the gamma regression which requires non-negative values. We undertook five separate sensitivity analyses to test the model structure, including an offset of +$10,000, an offset of +$100,000, excluding all negative excess costs, setting negative excess costs to $1, and regression models using lognormal regression (see Supporting Information). We also undertook sensitivity analyses excluding participants with costs among the highest and lowest 1% and 5%, to test for differences in the associations without potentially extreme values, along with setting year of diagnosis as a continuous variable to test for cost trends over time, and using a diagnosis up-to-/post-2013 binary variable to test for differences brought on by changes in available/subsidised treatments. We also tested the inclusion of cause of death (melanoma/skin cancer or other cause) in the regression model for the terminal phase, based on the subset of people dying to December 2019 who had cause of death information available.

We estimated the proportions of participants having each melanoma-related treatment type separately for invasive and *in situ* diagnoses, up to June 2020 when all datasets were available. Targeted therapies and immunotherapies were also analysed to June 2023 (the end of MBS/PBS data availability).

Analyses were carried out using SAS v9.4 and graphs were generated in R v4.4.1 and Microsoft Excel. Ethical approval for the 45 and Up Study was provided by the University of NSW Human Research Ethics Committee (HC210602). Specific approval for this analysis was provided by the NSW Population and Health Services Research Ethics Committee (HREC/14/CIPHS/54).

## Results

### Study cohort

We included 8577 participants diagnosed with melanoma during 2006–2019 who were matched with 33,868 cancer-free control participants (Fig 2); 97% of those with melanoma had four matched controls, 3% had 1–3 matched controls. For all participants, the median age was 62 years, 56% were male, and just over half were living in major cities at baseline (Table D in S1 File). Participants with melanoma and control participants had similar levels of education, BMI and comorbidities, but a higher proportion of those with melanoma had fair/very fair skin, were born in Australia, had a family history of melanoma, and had private health insurance.

There were 3551 participants first diagnosed with invasive melanoma (10% of these had a subsequent diagnosis of *in situ* melanoma during the study period), with median age 69 years at diagnosis and median Breslow thickness 0.7 mm (see Table 1 for details). The other 5026 participants were first diagnosed with *in situ* melanoma (5% had a subsequent diagnosis of invasive melanoma during the study period), also with median age 69 at diagnosis. From 2009 to 2019, the number of additional participants diagnosed with invasive melanoma remained relatively stable at ~300 per year, while the number of additional *in situ* cases increased from ~300 to ~550 per year. At diagnosis, 85% of people with invasive melanoma had localised stage, 7% had regional spread, 4% had distant metastases and 4% had unknown stage, similar to the NSW-wide distribution for 2006–2020 [23]. Five-year all-cause survival was 92% for those with *in situ* melanoma and controls, and 83% for those with invasive melanoma (localised 88%, regional 62%, metastatic 28%).

**Table 1:** Characteristics of 45 and Up Study participants diagnosed with melanoma 2006-2019.

	Invasive melanoma		Melanoma *in situ*	
	n	%	n	%
No. of participants	3551	100%	5026	100%
Age at diagnosis (years)	*(Median 69)*	*(IQR 62–77)*	*(Median 69)*	*(IQR 62–76)*
45-59	652	18%	914	18%
60-69	1178	33%	1770	35%
70-79	1035	29%	1607	32%
≥ 80	686	19%	735	15%
Sex				
Female	1508	42%	2263	45%
Male	2043	58%	2763	55%
Stage at diagnosis				
Localised	3011	85%	N/A	
Regional	259	7%	N/A	
Distant metastases	128	4%	N/A	
Unknown	153	4%	N/A	
Year of diagnosis				
2006-2008	243	7%	186	4%
2009	279	8%	331	7%
2010	295	8%	306	6%
2011	316	9%	343	7%
2012	300	8%	360	7%
2013	296	8%	396	8%
2014	296	8%	420	8%
2015	291	8%	498	10%
2016	344	10%	496	10%
2017	290	8%	575	11%
2018	323	9%	539	11%
2019	278	8%	576	11%
Breslow thickness	*(Median 0.7)*	*(IQR 0.4–1.8)*		
> 0 – < 0.4mm	699	20%	N/A	
0.4 – < 0.6mm	481	14%	N/A	
0.6 – < 0.8mm	681	19%	N/A	
0.8–1 mm	386	11%	N/A	
> 1–2 mm	509	14%	N/A	
> 2–4 mm	341	10%	N/A	
> 4mm	247	7%	N/A	
Unknown/Not applicable	207	6%	5026	100%
Site of cancer				
Trunk	1179	33%	1661	33%
Upper limbs	922	26%	1282	26%
Lower limbs	682	19%	627	12%
Face/head/neck	652	18%	1443	29%
Other/unspecified	116	3%	13	0%
Histology				
Superficial spreading	1640	46%	608	12%
Nodular	398	11%	0	0%
Lentigo	434	12%	1888	38%
Other specified	163	5%	26	1%
No information	916	26%	2504	50%
Overall survival^a^				
1 year		96%		99%
2 years		92%		98%
5 years		83%		92%

*IQR: Inter-quartile range; N/A: not applicable.*

^*a*^ *Kaplan-Meier survival estimates, from diagnosis date. Numbers are not listed due to differing levels of potential follow-up time. Corresponding levels for controls, using the diagnosis date of their matched case: 1 year 99%, 2 years 97%, 5 years 92%.*

### Costs

**Fig 4:**
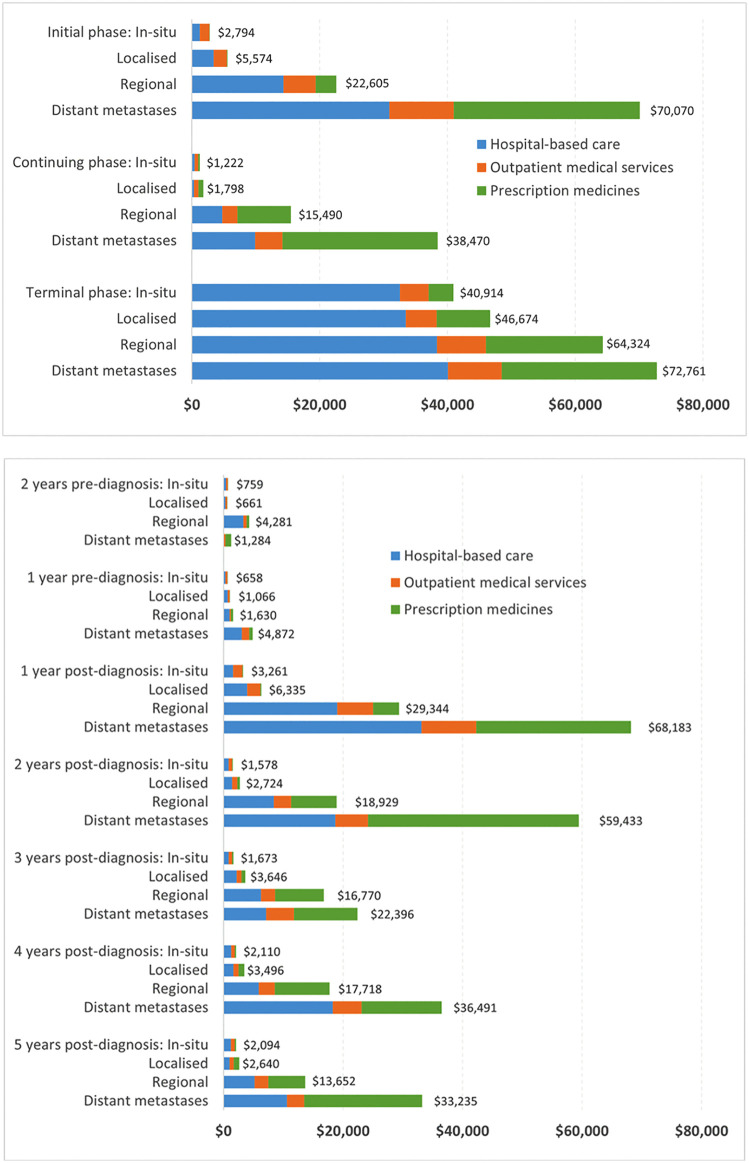
Mean excess costs for melanoma by stage of disease and source of costs, (A) by phase of care and (B) by year relative to diagnosis.

The mean per-person costs in the initial treatment phase increased substantially with melanoma stage, from $2794 for *in situ* melanoma, to $70,070 for distant metastases at diagnosis (Fig 4 and Tables E-G in S1 File). The mean annual costs in the continuing care phase had similar increases with stage, from $1222 (*in situ*) to $38,470 (distant metastases), and these annual costs were all greater than the corresponding costs in the year prior to diagnosis (e.g., *in situ* $658, distant metastases $4872). Per-person costs in the terminal phase were $64,324 for regional stage and $72,761 for distant metastases – for participants who died within 12 months of diagnosis, the mean costs were $61,794 and $54,141 respectively, and for those who died after ≥12 months, the estimates were $65,100 and $113,991 respectively. Only a very small proportion of participants with *in situ* (<1%) or localised melanoma (3%) died from melanoma (Table H in S1 File).

For invasive melanomas, hospital-based care comprised the majority of costs in the initial (57%) and terminal phases (66%), but prescription medicines comprised 50% of annual costs in the continuing care phase (Fig 4). Notably, in the continuing care phase, prescription medicines comprised 63% of costs for participants with metastatic stage and 54% for those with regional stage; for participants with localised stage, prescription medicines comprised 42%, with a further 41% due to MBS medical services. For participants first diagnosed with *in situ* melanoma, MBS services incurred the highest proportion of costs in the initial phase (53%) and continuing care phase (47%).

**Fig 5:**
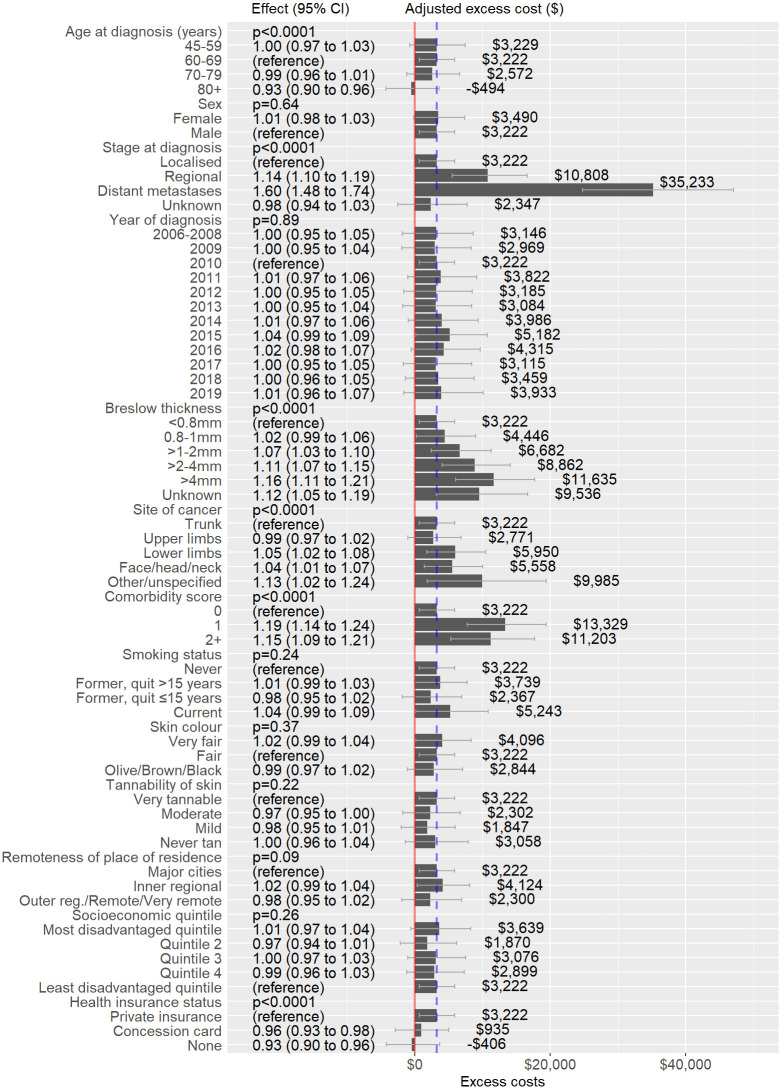
Association between participants’ characteristics and costs in the initial treatment phase for participants with invasive melanoma. *CI: confidence interval; reg.: regional. Note: The dashed vertical line is the estimated excess cost for a participant with invasive melanoma* ***who is in the reference category for all characteristics*** *(i.e., $3222 for a male aged 60-69 with localised stage, etc.). For each category of a characteristic, the estimate shown reflects the adjusted excess cost for a participant with all other characteristics in the reference category. To allow regression including negative excess costs, models were constructed using an offset of +$50,000; this offset was then deducted to obtain the adjusted estimates shown in the figure. See supporting information for estimates for continuing and terminal phases and estimates for in situ melanoma*.

Among participants with invasive melanoma, after adjusting for a range of characteristics, higher costs in the initial treatment and continuing care phases were most strongly associated with more advanced stage of disease at diagnosis (Fig 5, Fig B and C in S1 File). Higher costs were also associated with the presence of comorbidities and poorer self-reported health, thicker melanomas, having private health insurance, cancer site (lower limbs and face/head/neck vs trunk), and younger age at diagnosis. Higher costs in the terminal phase were mainly associated with younger age, with a trend of higher costs in more recent years; for those with cause of death information, there were higher costs for people who died from melanoma or skin cancer compared to other causes (see Supporting Information). There was no other association between year and costs by phase or invasive/*in situ* melanoma. For participants with *in situ* melanoma, higher costs in the initial and continuing phases were strongly associated with comorbidities and younger age at diagnosis, with slightly higher initial phase costs for melanomas of the face/head/neck. Excluding the highest and lowest 1% or 5% of values generally had little impact on these associations.

### Treatment

Similar proportions of those with *in situ* and localised melanoma had a record of a skin biopsy (78% and 74% respectively), cryotherapy/curettage/laser ablation/other destruction of a skin lesion (both 63%), and skin flap repair/graft (37% and 44%) (Table 2). The proportions for participants with metastatic melanomas were considerably lower, at 30%, 23% and 23% respectively. Sentinel lymph node biopsies were more common for invasive melanomas (16% vs 2% of *in situ*; highest: 35% of regional stage), as was chemotherapy (10% vs 3%; highest: 52% of metastatic) and radiotherapy (11% vs 6%; highest: 44% of metastatic). The vast majority of participants with melanoma had an MBS record of a melanoma excision (invasive 83%, *in situ* 89%; lowest: 25% of metastatic) or any malignant skin excision (invasive 91%, *in situ* 95%; lowest: 39% of metastatic; also recorded for 29% of controls).

**Table 2:** Procedures recorded for participants with melanoma diagnosed 2006-2019 by stage of disease at diagnosis, and their matched controls.

Procedure (from 1 month pre-diagnosis to June 2020)	*In situ*		Localised stage		Regional stage		Distant metastases		Unknown stage		*Controls*
	n	%	n	%	n	%	n	%	n	%	*%*
Number of participants	5026		3011		259		128		153		33,868
Skin biopsy	3907	78%	2215	74%	174	67%	38	30%	114	75%	35%
Skin flap repair/graft	1846	37%	1316	44%	160	62%	30	23%	68	44%	11%
Sentinel lymph node biopsy	100	2%	455	15%	91	35%	16	13%	16	10%	0%
Cryotherapy/curettage/laser ablation/ other destruction of a skin lesion											
Malignant (recorded in MBS only)	1393	28%	881	29%	67	26%	10	8%	50	33%	12%
Any skin lesion	3182	63%	1888	63%	128	49%	29	23%	93	61%	36%
Skin excision: melanoma (MBS only)	4456	89%	2626	87%	184	71%	32	25%	116	76%	1%
Skin excision: any malignant (MBS only)	4751	95%	2823	94%	214	83%	50	39%	132	86%	29%
Any skin excision	4946	98%	2970	99%	247	95%	63	49%	139	91%	38%
Chemotherapy	175	3%	208	7%	73	28%	66	52%	23	15%	N/A
Radiotherapy	307	6%	225	7%	83	32%	56	44%	27	18%	N/A
Immunotherapy^a^	29	1%	77	3%	38	15%	40	31%	9	6%	N/A
-including PBS to June 2023	70	1%	126	4%	45	17%	41	32%	11	7%	N/A
Targeted therapy^b^	n.r.	n.r.	17	1%	17	7%	10	8%	n.r.	n.r.	N/A
Immunotherapy or targeted therapy	29	1%	86	3%	46	18%	43	34%	11	7%	N/A
-including PBS to June 2023	73	1%	135	4%	54	21%	n.r.	n.r.	n.r.	n.r.	N/A
Test for BRAF V600 mutation^c^	52	1%	160	5%	64	24%	38	30%	15	10%	N/A

^a^ *includes pembrolizumab, ipilimumab, nivolumab (immune checkpoint inhibitors).*

^b^ *includes vemurafenib/cobimetinib, dabrafenib/trametinib, encorafenib/binimetinib (n = 0) (BRAF/MEK inhibitors); there were very few additional records when including PBS data to June 2023.*

^c^ *MBS item 73,336 testing for BRAF V600 mutation for dabrafenib access (first recorded in 2013), to June 2023.*

*MBS: Medicare Benefits Schedule; N/A: not applicable; n.r.: Not reported due to small numbers in the cell or from adjacent cell; PBS: Pharmaceutical Benefits Scheme.*

*Procedure/item codes for cryotherapy/curettage/laser/other destruction of lesion were combined as they are not specific to each procedure and can cover more than one of these procedures.*

A small proportion of participants first diagnosed with *in situ* or localised melanoma had a record of immunotherapy and/or targeted therapy. Among participants with more advanced melanoma, there was a substantial increase over time in the proportion who received immunotherapy and/or targeted therapy: from 7% for metastatic and 11% for regional stage (9% combined) for those diagnosed prior to 2014, up to 66% and 30% respectively (41% combined) for the 195 participants first diagnosed during 2014–2019. The most common therapies recorded for these 195 participants were the immune checkpoint inhibitors pembrolizumab (22%), ipilimumab (10%) and nivolumab (5%; 32% received at least one of these three), and targeted therapies dabrafenib and/or trametinib (10%), while 45% had a molecular test recorded for the BRAF V600 mutation as part of eligibility testing for dabrafenib. For the 80 participants with advanced melanoma diagnosed 2014–2019 who had immunotherapy and/or targeted therapy, the mean costs were $75,632 in the initial phase, $69,096 per year in the continuing care phase and $135697 in the terminal phase, compared with $17,622, $3321 and $35,612 respectively for the 115 participants with advanced melanoma diagnosed 2014–2019 who did not have these treatments.

## Discussion

In this large, population-based study of people diagnosed with melanoma in Australia, we found that health system costs were much higher for those with more advanced melanoma at diagnosis, both in the initial treatment phase and the continuing care phase, even after adjusting for confounding factors. Almost half of all study participants diagnosed with regional disease or metastatic melanoma after 2013 had a record of immunotherapy and/or targeted therapy. The average costs for participants receiving these specific treatments were approximately $60,000 per year higher than costs for participants with a similar stage but not receiving these therapies, reflecting the potential for additional costs for the treatment itself and any monitoring, investigations, tests, and adverse events relating to the treatment. These results emphasise the importance of prevention and early detection of melanoma, for both health and economic benefits.

The trend of increasing costs with more advanced stage melanoma matches other Australian and international studies [29]. A 2022 bottom-up modelling study reported that for people with melanoma, the related health system costs in the first year after diagnosis ranged from $644 for *in situ*/stage I up to $100,725 for unresectable stage III/IV [15]. A 2017 study estimated 1-year post-diagnosis costs of $1681 for *in situ*/stage I/II, $37,729 for stage III resectable, and $115,109 for unresectable stage III/IV, with 3-year post-diagnosis costs of $187,720 for the latter group [30]. Our results had different stage and phase of care groupings to both studies so the results cannot be directly compared, but the lowest and highest cost estimates were comparable and the strong trend of increasing costs with stage is clear.

The large difference in costs for regional/metastatic versus localised stage for melanoma was greater than the stage-based differences previously reported for lung and colorectal cancers in Australia [31,32]. However, the lung cancer study was limited to costs up to 2016, prior to PBS listing of newer therapies. A subsequent study incorporating those results for lung cancer and alongside costs of newer therapies estimated that although people receiving immunotherapy and/or targeted therapies only represent a small proportion of lung cancers, the introduction of these therapies resulted in a doubling of total lung cancer care costs [33]. Similarly, given current treatment patterns, even if only a small proportion of melanomas are currently diagnosed at advanced stage, their treatment costs have a disproportionate impact on the overall melanoma healthcare costs. Thus, there could be substantial treatment cost savings from a reduction in advanced disease through potential future screening programs. We found there was increasing uptake of immunotherapies and targeted therapies for participants diagnosed with more advanced melanoma. More recent trials have shown benefits of immunotherapy for people with stage II disease, so it is plausible that the proportion of melanomas treated in this way will increase, further increasing costs overall [13]. On an individual-level, we also found that higher costs were associated with the presence of comorbidities, having thicker melanoma, and having private health insurance, while there were lower costs for those aged 80+ at diagnosis. However, all of these differences were relatively minor compared with the stage-based cost differences.

A very low proportion of participants with *in situ* or localised melanoma died from melanoma. Thus, the terminal phase costs for participants with *in situ* or localised melanoma largely represents end-of-life healthcare costs for non-melanoma causes of death. For participants with cause of death information available, terminal phase costs were higher for those who died from melanoma/skin cancer, potentially due to greater use of more expensive treatments such as immunotherapy and targeted therapy towards the end of life. The rare instances of immunotherapy or targeted therapy for people first diagnosed with *in situ* or localised melanoma are likely due to having a subsequent advanced invasive primary melanoma or progression of disease, as these therapies would not be given for *in situ*/localised disease, and multiple primaries are relatively common [34].

Our previous costing study including the 10 most common cancer types found that participants with melanoma had among the lowest health system costs per person [14]. However, with the large number of new melanoma cases each year, there is still a substantial cost to the health system. Based on all new cases of melanoma in Australia 2019−2023, our results suggest $500 million in health system costs in 2023 for people with invasive melanoma alive in 2023, with a further $290 million for people with *in situ* melanoma (Table I in S1 File). An Australian government report estimated that the healthcare cost of invasive melanoma in 2023 was $425 million [35], suggesting that even after inflation, the participants in our study with melanoma might have higher than expected healthcare costs. Furthermore, our estimate does not include the potentially substantial continuing care costs for people living with melanoma diagnosed pre-2019. The Australian government report also estimated the cost of non-melanoma skin cancers in 2023 was $1.83 billion, an increase of 21% from the $1.52 billion reported in 2021 [36].

Notably, we found mean excess costs of ~$1000 per person with melanoma in each of the two years before their diagnosis. The excess costs method does not identify specific healthcare items, such as screening or specialist visits, rather, it is a measure of the overall costs of additional healthcare use. Even allowing for some diagnostic costs, this finding suggests that in our cohort and potentially across Australia, people diagnosed with melanoma might have a higher rate of interacting with the health system in general compared to those without melanoma. If that higher rate of health system interaction continues post-diagnosis, it might also inflate the cost estimates for different treatment phases in this study (with the potential inflation by stage and phase accounting for approximately 30% of initial phase costs and approximately 50% of continuing phase costs estimated for participants with *in situ* and localised disease, and approximately 10%−15% of the initial and continuing phase costs estimated for participants with regional and distant disease at diagnosis). The increased rate of health system interaction could also contribute to potential overdiagnosis of melanomas.

The potential over-diagnosis or over-detection of skin cancers, particularly *in situ* melanomas [37], is also a key consideration for screening programs. People already interacting with the health system for other reasons might be more likely to take part in skin cancer screening, especially if recommended by their physician. There is an effective treatment for early-stage disease (surgical excision) and an effective diagnostic test (visual skin examination with dermoscopy with histopathological review of an excision biopsy), and there is already considerable opportunistic screening taking place, with approximately one-third of Australians reporting having their skin checked by a doctor in the previous 12 months [38]. The potential for over-diagnosis can be at least partially addressed by implementing a risk-based screening program, which could reduce unnecessary tests among people less likely to have invasive melanoma. While an organised population-wide melanoma screening program is not currently in place or recommended in Australia, the evidence for risk-tailored screening is being continually evaluated [39]. To reduce future cancer burden, a recent systematic review found that allocating health system resources to primary prevention of skin cancer is highly favourable from both population health and economic perspectives [40], and another review highlighted the equity-related benefits of prevention [41].

This study has some limitations. The 45 and Up Study cohort is not directly representative of the whole Australian population, as study participants are generally healthier and have higher educational levels [42], and are potentially more frequent users of preventive health services. This could bias our findings towards higher costs and uptake of targeted therapies and immunotherapies compared to the general population. There were no major differences in costs by geographical area, but there were differences for people aged 80+ (oversampled in this cohort), and we did not have any participants aged <45, noting melanoma incidence is low in the latter group and has been declining in recent years [3]. Nevertheless, the stage distribution was similar to all of NSW [23], as was survival by stage, with key results mainly reported by stage, and any combined results based on weighted data to match the NSW stage distribution, which represents approximately one-third of all cancers in Australia. Within the study cohort, participants with melanoma had fairer skin than controls, were more commonly Australian-born, with family history of melanoma and private health insurance. The first three characteristics are associated with melanoma risk, and the latter is potentially related to general healthcare utilisation and healthcare costs, with this difference possibly contributing to over-diagnosis but also subsequent higher costs. Having private health insurance has been found to be associated with more specialist consults and hospital services in patients with melanoma [43]. We considered matching by private health insurance, although given the complex relationship between health insurance and health service use it wasn’t clear if this would reduce confounding or introduce a new source of confounding.

Further, this is an observational study and is likely to have other unmeasured biases. For example, we were only able to analyse participants based on their first invasive melanoma diagnosis, and did not have subsequent primary/recurrence/progression data, so some people might have higher costs due to additional cancers that we could not identify, and we did not have information on patient treatment choices. Also, using the supplied summary stage groupings (localised, regional, distant metastases at diagnosis) does not line up exactly with the more detailed clinical staging categories from the American Joint Committee on Cancer [44] that determine treatment recommendations, potentially obscuring a more detailed picture of costs by clinical stage, particularly for non-localised disease. However, the NSW Cancer Registry is one of the few cancer registries in Australia that routinely makes staging information available for analysis, so being able to differentiate between spread of disease at this less granular level for such a large cohort is still a substantial step forward for evaluation of melanoma care in Australia.

The costs we used for targeted therapy and immunotherapy are based on PBS listed prices, and could differ substantially from the actual costs to the government due to confidential pricing arrangements with pharmaceutical companies [45]. We also have not included patients using ‘off label’ therapies that are not yet publicly funded, and therapies provided through participation in clinical trials. We did not have information on people first diagnosed with melanoma from 2020 onwards and there might have been changes in the treatment of advanced melanomas since then. We also assumed that private hospital costs had the same value as public hospital costs to the health system. We did not have information when people had interstate public hospital admissions, or records of care beyond that in admitted hospitalisations, public emergency departments or through MBS/PBS, such as community-based palliative care or residential aged care. Out-of-pocket healthcare costs incurred by individuals were outside the scope of this study. Costs are reported in 2019 Australian dollars, to convert these to 2024 dollars based on the Australian Health Index alone would mean increasing all costs by approximately 20% [21].

A substantial proportion of people with melanoma had negative excess costs in certain time periods (Table F in S1 File). Negative excess costs occur when a person with cancer has lower costs than the average costs for their matched controls during that time period, which can occur due to random variation in healthcare costs for controls (who may also experience non-melanoma health conditions leading to high costs). We have previously used the excess costs method for several cancer types and have found that people with melanoma generally have lower excess costs than people with other cancer types [14,31,32]. This means that expected random fluctuations around mean excess cost values are more likely to lead to some instances of negative excess costs for melanoma case-control groupings, but these are still valid excess cost observations, and the large sample and use of multiple matched controls help to smooth out any potential impact of random variation and instances of negative excess costs. Finally, the excess cost approach used here does not incorporate potential future treatment options, although the study benefits from its comprehensive capture of existing health system use.

A key strength of this study was the ability to quantify differences by stage, for a relatively recent cohort, and we found substantial stage-based differences in costs, treatment and survival. Treatment recommendations have been revised, especially since 2013, and we used more recent data to investigate the uptake of new therapies. The study used a large longitudinal cohort with a population-based sampling frame and extensive information on participants, so we could examine associations between costs and a wide range of participants’ characteristics. By using the excess cost method, we have included the healthcare cost impact of downstream adverse events. We have also addressed a gap in knowledge about the associations between health system costs and various characteristics of individuals, such as age, thickness of invasive melanomas, remoteness of residence and private health insurance status.

Overall, this study clearly quantifies differences in melanoma-related costs by stage in Australia. The vast differences in costs by stage highlight the importance of early detection and prevention. The results give a real-world context of healthcare costs for people with *in situ* and localised disease, and the cost implications for people diagnosed with melanoma at regional and metastatic stage. These relatively recent data provide estimates that can help to evaluate the costs and benefits of a potential targeted national skin cancer screening program, to determine the most effective and cost-effective strategies for reducing the future burden of melanoma.

## Supporting information

S1 File Detailed descriptive statistics, results, and figures. (DOCX)
