# Correction: Health system costs and systemic treatment patterns by disease stage for 8577 people diagnosed with melanoma in New South Wales, Australia 2006–2019

**DOI:** 10.1371/journal.pone.0361490

**Published:** 2026-10-23

**Authors:** David E. Goldsbury, Anne E. Cust, Caroline Watts, Alison Pearce, Philip Haywood, Louisa G. Collins, Preston Ngo, Kirstie McLoughlin, Michael Caruana, Karen Canfell, Julia Steinberg, Marianne F. Weber

The Funding statement for this article is incorrect. The correct Funding statement is as follows: This work was supported by Cancer Council NSW, through funding to the Daffodil Centre. JS is a recipient of a Cancer Institute NSW Career Development Fellowship (2022/CDF1154). AEC was supported by a NHMRC Investigator Grant #2008454.
