# Supplementary material for: Health system costs and systemic treatment patterns by disease stage for 8577 people diagnosed with melanoma in New South Wales, Australia 2006–2019
Source: PLoS One. 2026 Jul 13;21(7):e0353408. doi: 10.1371/journal.pone.0353408 (PMC13362142; doi:10.1371/journal.pone.0353408)
Supplement: S1 File — (DOCX) [file pone.0353408.s001.docx]

**Supporting Information S1 File**

**Health system costs and systemic treatment patterns by disease stage for 8577 people diagnosed with melanoma in New South Wales, Australia 2006-2019**

**Fig A.** Timeline of approvals for government subsidising and inclusion on Pharmaceutical Benefits Scheme in Australia for drugs to treat melanoma.


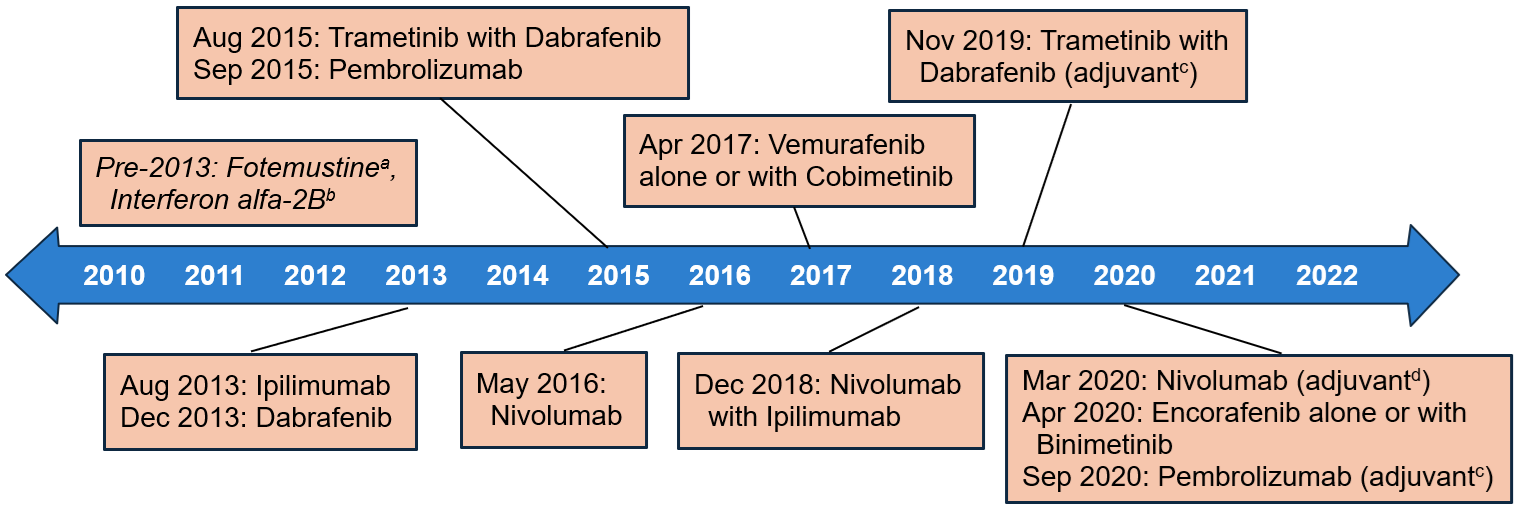


*Note: All are for unresectable stage III/IV melanoma, unless otherwise stated.*

*^a^ Fotemustine was listed for “metastatic melanoma” in 2005 and was removed from the PBS in August 2022*

*^b^ Interferon alfa-2B was listed for “metastatic melanoma” in 2000 and was removed from the PBS in June 2018.*

*^c^ Adjuvant treatment for resected stage IIIB-IIID melanoma*

*^d^ Adjuvant treatment for resected stage IIIB-IV melanoma*

**Table A**. Codes used to identify melanoma-related procedures.

| **Treatment type** | **Data type** | **Codes/items** |
| --- | --- | --- |
| Skin biopsy | APDC procedure | 30071-00 |
|  | MBS item | 30071 |
| Skin flap repair/ graft | APDC procedure | 45200,45201,45202,45203,45206,45207,45209,45212,45215,45218,45221, 45224,45227,45230,45233,45236,45239,45400-00,45403-00, 45439, 45442, 45445,45448, 45451-0x, 45451-24,45451-24,45451-25,45451-26,45451-27 |
|  | MBS item | 45200,45201,45202,45203,45206,45207,45209,45212,45215,45218,45221, 45224,45227,45230,45233,45236,45239,45400,45403, 45439,45442, 45445,45448, 45451 |
| Sentinel lymph node biopsy | APDC procedure | 30300,96243 |
|  | MBS item | 30299,30300,30301,30302,30303,30311 |
| Cryotherapy / curettage / laser / other destruction of skin lesion^a^ | APDC procedure | 30071-00, 30190-00, 30192-00, 30195-00, 30195-01, 30195-02, 30195-03, 30195-04, 30195-05, 30195-06, 30195-07, 30196-00, 30196-01, 30197-00, 30197-01, 30202-00, 30203-00, 30205-00, 30205-01 |
|  | MBS item | Malignant: 30196,30197,30202,30203,30205  Others: 30190,30191,30192,30195 |
| Excision of skin lesion | APDC procedure (including all sub-codes) | 31000,31200,31205-00,31210,31215,31220,31225,31230,31235,31240,31250, 31255 to 31258,31260 to 31263, 31265 to 31268, 31270 to 31273, 31275 to 31278, 31280 to 31283, 31285 to 31288, 31290 to 31293, 31295, 31300, 31305, 31310, 31315, 31320, 31325, 31356-31370 |
|  | MBS item | Melanoma: 31300 to 31335, 31371 to 31376  Other malignant: 31255 to 31299, 31356, 31358, 31359, 31361, 31363, 31365, 31367, 31369  Others: 31000 to 31005, 31200, 31205, 31206, 31210, 31215, 31216, 31220, 31221, 31225, 31230, 31235, 31240, 31245, 31250, 31357, 31360, 31362, 31364, 31366, 31368, 31370 |
| Chemotherapy | APDC procedure (including all sub-codes) | 13915,13918,13921,13924,13927, 90760-00, 90767-00, 90768-00, 96199-00, 96199-09, 96200-00, 96201-00, 96203-00, 96204-00 |
|  | APDC diagnosis | Z51.1, Z51.2 |
|  | MBS item | 13915,13918,13921,13924,13927,13930,13933,13936,13945 |
|  | PBS ATC code | Fotemustine: L01AD05 |
| Radiotherapy | APDC procedure | 15000,15003,15100-00,15103,15203-00,15204-00,15207-00,15208-00, 15215, 15224-00, 15227, 15230, 15233, 15239-00, 15242, 15245, 15251, 15254-00, 15257, 15260, 15266, 15269-00, 15272, 15500, 15503 15506-01,15506-02, 15509, 15512, 15515, 15518, 15521, 15524, 15527, 15530, 15533, 15550-00, 15556, 15559, 15562, 15600, 15700, 15705, 15710, 15715, 90765-00, 90765-01, 90765-02, 90765-03 |
|  | APDC diagnosis | Z51.0 |
|  | MBS item | 15000-15399, 15500-15600, 15700-15899 |
| Immunotherapy | PBS ATC code | Immune checkpoint inhibitors  Ipilimumab: L01FX04, L01XC11  Nivolumab: L01FF01, L01XC17  Pembrolizumab: L01FF02, L01XC18 |
| Targeted therapy | PBS ATC code | BRAF/MEK inhibitors  Vemurafenib: L01EC01, L01XE15  Cobimetinib: L01EE02, L01XE38  Dabrafenib: L01EC02, L01XE23  Trametinib: L01EE01, L01XE25  Encorafenib: L01EC03, L01XE46  Binimetinib: L01EE03, L01XE41 |
| Molecular testing | MBS item | 73336 (BRAF V600 mutation for access to dabrafenib) |

*^a^ Combined as the procedure/item codes are not specific to individual procedures, can cover multiple.*

*APDC: Admitted Patient Data Collection; ATC: Anatomical Therapeutic Chemical; MBS: Medicare Benefits Schedule; PBS: Pharmaceutical Benefits Scheme.*

**Table B**. Categorisations from 45 and Up Study questions and other sources.

| **Characteristic / Category** | **[Source] Question / Response** |
| --- | --- |
| Health insurance | *[45 and Up Study baseline] Which of the following do you have? (excluding Medicare)* |
| Private health insurance | Private health insurance – with extras; Private health insurance – without extras |
| Department of Veterans’ Affairs | Department of Veterans’ Affairs white or gold card |
| Concession card | Health care concession card |
| None | None of these |
| Skin colour | *[45 and Up Study baseline] What best describes the colour of the skin on the inside of your upper arm, that is your skin colour without any tanning?* |
| Very fair | Very fair |
| Fair | Fair |
| Light olive | Light olive |
| Dark olive / Brown / Black | Dark olive; Brown; Black |
| Skin response to time in the sun (tannability) | *[45 and Up Study baseline] What would happen if your skin was repeatedly exposed to bright sunlight during summer without any protection? Would it:* |
| Get very tanned | Get very tanned? |
| Get moderately tanned | Get moderately tanned? |
| Get mildly tanned | Get mildly or occasionally tanned? |
| Never tan, only freckle | Never tan, or only get freckled? |
| Cancer site | *[NSW Cancer Registry] ICD-10 topography* |
| Trunk | C43.5 (invasive), D03.5 (in situ) |
| Upper limbs | C43.6 (invasive), D03.6 (in situ) |
| Lower limbs | C43.7 (invasive), D03.7 (in situ) |
| Face/head/neck | C43.0-C43.4 (invasive), D03.0-D03.4 (in situ) |
| Other/unspecified | All others starting with C43 (invasive) or D03 (in situ) |
| Cancer histology | *[NSW Cancer Registry] ICD-O-3 morphology* |
| Superficial spreading | 87433 (invasive), 87433 (in situ) |
| Nodular | 87213 (invasive) |
| Lentigo | 87423 (invasive), 87422 (in situ) |
| No information | 87203 (invasive), 87202 (in situ) |
| Other specified | All others ending in “3” (invasive), all others ending in “2” (in situ) |

*ICD-10: International Statistical Classification of Diseases and Related Health Problems, Tenth Revision*

*ICD-O-3: International Classification of Diseases for Oncology, Third Edition*

More details: <https://www.saxinstitute.org.au/solutions/45-and-up-study/use-the-45-and-up-study/data-and-technical-information>

**Table C**. Diagnosis codes used to identify comorbidities in the Admitted Patient Data Collection.

| Condition | Diagnosis codes (ICD-10-AM) | Weight |
| --- | --- | --- |
| Acute myocardial infarction | I21, I22, I25.2 | 0 |
| Congestive heart failure | I50 | 2 |
| Peripheral vascular disease | I71, I73.9, I79.0, R02, Z95.8, Z95.9 | 0 |
| Cerebral vascular accident | I60-I66, I69, G46, G45.0-G45.2, G45.4, G45.8, G45.9, I67.0-I67.2, I67.4-I67.9, I68.1, I68.2, I68.8 | 0 |
| Dementia | F00, F01, F02, F05.1 | 2 |
| Pulmonary disease | J40-J47, J60-J67 | 1 |
| Connective tissue disorder | M32, M34, M05.0-M05.3, M05.8, M05.9, M06.0, M06.3, M06.9, M33.2, M35.3 | 1 |
| Peptic ulcer | K25, K26, K27, K28 | 0 |
| Liver disease | K73, K70.2, K70.3, K71.7, K74.0, K74.2, K74.3, K74.4, K74.5, K74.6 | 2 |
| Diabetes without complications | E10.9, E11.9, E13.9, E14.9, E10.1, E11.1, E13.1, E14.1, E10.5, E11.5, E13.5, E14.5 | 0 |
| Diabetes with complications | E10.2, E11.2, E13.2, E14.2, E10.3, E11.3, E13.3, E14.3, E10.4, E11.4, E13.4, E14.4 | 1 |
| Paraplegia | G81, G04.1, G82.0, G82.1, G82.2 | 2 |
| Renal disease | N05.2-N05.6, N07.2, N07.3, N07.4, N03, N01, N18, N19, N25 | 1 |
| Severe liver disease | K72.9, K76.6, K76.7, K72.1 | 4 |
| AIDS/HIV | B20, B21, B22, B23, B24 | 4 |

*ICD-10-AM: International Statistical Classification of Diseases and Related Health Problems, 10^th^ Revision, Australian Modification. Codes from 2011 Quan et al. modification of the Charlson Comorbidity Index* (1)*. Shows some conditions with zero weight that were in the original Charlson index. Cancers were not included as comorbidities.*

**Table D**. Sociodemographic characteristics of cases and matched controls for invasive and *in situ* melanomas diagnosed 2006-2019.

|  | Invasive cases | | Invasive controls | | *In situ* cases | | *In situ* controls | |
| --- | --- | --- | --- | --- | --- | --- | --- | --- |
|  | n | % | n | % | n | % | n | % |
| Total number | 3551 |  | 13993 |  | 5026 |  | 19875 |  |
| Age at baseline (years) |  |  |  |  |  |  |  |  |
| *Median age (IQR)* | *63* | *(56-71)* | *63* | *(56-71)* | *62* | *(55-68)* | *61* | *(55-68)* |
| 45-59 | 1346 | 38% | 5354 | 38% | 2112 | 42% | 8393 | 42% |
| 60-69 | 1150 | 32% | 4628 | 33% | 1793 | 36% | 7138 | 36% |
| 70-79 | 765 | 22% | 2948 | 21% | 876 | 17% | 3389 | 17% |
| ≥80 | 290 | 8% | 1063 | 8% | 245 | 5% | 955 | 5% |
| Sex |  |  |  |  |  |  |  |  |
| Female | 1508 | 42% | 5958 | 43% | 2263 | 45% | 8995 | 45% |
| Male | 2043 | 58% | 8035 | 57% | 2763 | 55% | 10880 | 55% |
| Remoteness of place of residence at baseline |  |  |  |  |  |  |  |  |
| Major cities | 1789 | 50% | 7105 | 51% | 2635 | 52% | 10416 | 52% |
| Inner regional | 1401 | 39% | 5480 | 39% | 1914 | 38% | 7553 | 38% |
| Outer regional/Remote/Very remote | 361 | 10% | 1408 | 10% | 477 | 9% | 1906 | 10% |
| Area-level socioeconomic quintile at baseline |  |  |  |  |  |  |  |  |
| Most disadvantaged quintile | 705 | 20% | 2848 | 20% | 854 | 17% | 3656 | 18% |
| Quintile 2 | 728 | 21% | 3038 | 22% | 1007 | 20% | 4023 | 20% |
| Quintile 3 | 651 | 18% | 2683 | 19% | 954 | 19% | 3787 | 19% |
| Quintile 4 | 657 | 19% | 2323 | 17% | 902 | 18% | 3463 | 17% |
| Least disadvantaged quintile | 776 | 22% | 2962 | 21% | 1260 | 25% | 4769 | 24% |
| *Missing* | 34 | 1% | 139 | 1% | 49 | 1% | 177 | 1% |
| Health insurance status at baseline |  |  |  |  |  |  |  |  |
| Private insurance | 2475 | 70% | 9006 | 64% | 3720 | 74% | 13370 | 67% |
| Concession card | 586 | 17% | 2631 | 19% | 668 | 13% | 3267 | 16% |
| None | 423 | 12% | 2090 | 15% | 569 | 11% | 2923 | 15% |
| *Missing* | 67 | 2% | 266 | 2% | 69 | 1% | 315 | 2% |
| Smoking status at baseline |  |  |  |  |  |  |  |  |
| Never | 2140 | 60% | 8502 | 61% | 3111 | 62% | 12374 | 62% |
| Former, quit >15 years | 931 | 26% | 3648 | 26% | 1304 | 26% | 5150 | 26% |
| Former, quit ≤15 years | 316 | 9% | 1228 | 9% | 415 | 8% | 1596 | 8% |
| Current | 164 | 5% | 615 | 4% | 196 | 4% | 755 | 4% |
| Self-reported health at baseline |  |  |  |  |  |  |  |  |
| Excellent | 559 | 16% | 2183 | 16% | 870 | 17% | 3336 | 17% |
| Very good | 1386 | 39% | 5299 | 38% | 2101 | 42% | 7610 | 38% |
| Good | 1130 | 32% | 4466 | 32% | 1497 | 30% | 6278 | 32% |
| Fair/Poor | 358 | 10% | 1577 | 11% | 423 | 8% | 2028 | 10% |
| *Missing* | 118 | 3% | 468 | 3% | 135 | 3% | 623 | 3% |
| Charlson comorbidity score^a^ |  |  |  |  |  |  |  |  |
| 0 | 3170 | 89% | 12564 | 90% | 4600 | 92% | 18099 | 91% |
| 1 | 206 | 6% | 754 | 5% | 276 | 5% | 971 | 5% |
| ≥2 | 175 | 5% | 675 | 5% | 150 | 3% | 805 | 4% |
| Body Mass Index at baseline |  |  |  |  |  |  |  |  |
| Normal/Underweight (<25kg/m^2^)^b^ | 1141 | 32% | 4766 | 34% | 1707 | 34% | 7114 | 36% |
| Overweight (25-<30kg/m^2^) | 1456 | 41% | 5639 | 40% | 2099 | 42% | 7826 | 39% |
| Obese (≥30kg/m^2^) | 793 | 22% | 2820 | 20% | 990 | 20% | 3987 | 20% |
| *Missing* | 161 | 5% | 768 | 5% | 230 | 5% | 948 | 5% |
| Highest education level attained |  |  |  |  |  |  |  |  |
| University degree or higher | 808 | 23% | 3336 | 24% | 1234 | 25% | 5049 | 25% |
| Trade/Certificate/Diploma/Apprenticeship | 1213 | 34% | 4604 | 33% | 1730 | 34% | 6678 | 34% |
| Higher school certificate | 306 | 9% | 1292 | 9% | 441 | 9% | 1805 | 9% |
| School certificate | 838 | 24% | 2960 | 21% | 1149 | 23% | 4015 | 20% |
| No school certificate | 343 | 10% | 1563 | 11% | 422 | 8% | 2072 | 10% |
| *Missing* | 43 | 1% | 238 | 2% | 50 | 1% | 256 | 1% |
| Country of birth |  |  |  |  |  |  |  |  |
| Australia | 3051 | 86% | 10327 | 74% | 4358 | 87% | 14682 | 74% |
| Other | 474 | 13% | 3552 | 25% | 635 | 13% | 5009 | 25% |
| *Missing* | 26 | 1% | 114 | 1% | 33 | 1% | 184 | 1% |
| Marital status at baseline |  |  |  |  |  |  |  |  |
| Married/de facto | 2784 | 78% | 10898 | 78% | 4074 | 81% | 15598 | 78% |
| Other | 752 | 21% | 3018 | 22% | 923 | 18% | 4156 | 21% |
| *Missing* | 15 | 0% | 77 | 1% | 29 | 1% | 121 | 1% |
| Skin colour |  |  |  |  |  |  |  |  |
| Very fair | 754 | 21% | 1894 | 14% | 943 | 19% | 2830 | 14% |
| Fair | 2174 | 61% | 7721 | 55% | 3086 | 61% | 10823 | 54% |
| Light olive | 544 | 15% | 3658 | 26% | 889 | 18% | 5258 | 26% |
| Dark olive/Brown/Black | 38 | 1% | 535 | 4% | 67 | 1% | 732 | 4% |
| *Missing* | 41 | 1% | 185 | 1% | 41 | 1% | 232 | 1% |
| Skin response to time in sun (tannability) |  |  |  |  |  |  |  |  |
| Get very tanned | 641 | 18% | 3995 | 29% | 1028 | 20% | 5615 | 28% |
| Get moderately tanned | 1417 | 40% | 5781 | 41% | 2079 | 41% | 8207 | 41% |
| Get mildly tanned | 918 | 26% | 2827 | 20% | 1251 | 25% | 4015 | 20% |
| Never tan, only freckle | 510 | 14% | 1126 | 8% | 589 | 12% | 1643 | 8% |
| *Missing* | 65 | 2% | 264 | 2% | 79 | 2% | 395 | 2% |
| Family history of melanoma (baseline) | 475 | 13% | 1112 | 8% | 671 | 13% | 1686 | 8% |
| Self-reported melanoma (baseline) | 166 | 5% | 319 | 2% | 219 | 4% | 431 | 2% |
| Self-reported other skin cancer (baseline) | 1496 | 42% | 3537 | 25% | 2083 | 41% | 5075 | 26% |
| Self-reported any skin cancer (baseline) | 1587 | 45% | 3702 | 26% | 2187 | 44% | 5319 | 27% |
| Self-reported skin cancer removal (baseline) | 1480 | 42% | 3486 | 25% | 2077 | 41% | 4910 | 25% |

*^a^ Controls used the diagnosis date of their matched case as the index date for the 5-year comorbidity lookback period.*

*^b^ “Underweight” (<18.5kg/m^2^) accounted for ~1% of each case/control group.*

*IQR: inter-quartile range.*

**Table E**. Mean excess per-person costs for people with melanoma, by source and stage at diagnosis.

| **Stage at diagnosis /**  Cost / Source | **>1-2 years pre-diagnosis** | **1 year pre-diagnosis** | **Initial phase** | **Continuing phase (per year)** | **Terminal phase** |
| --- | --- | --- | --- | --- | --- |
| ***In situ* (*n*)** | *5001* | *5026* | *4706* | *4091* | *427* |
| Mean excess cost per person | $759 | $658 | $2,794 | $1,222 | $40,914 |
| Hospital-based care (%) | 57% | 50% | 44% | 33% | 80% |
| MBS (%) | 37% | 48% | 53% | 47% | 11% |
| PBS (%) | 5% | 2% | 3% | 20% | 9% |
| **Localised (*n*)** | *2981* | *3011* | *2832* | *2534* | *425* |
| Mean excess cost per person | $661 | $1,066 | $5,574 | $1,798 | $46,674 |
| Hospital-based care (%) | 47% | 64% | 61% | 17% | 72% |
| MBS (%) | 39% | 34% | 38% | 41% | 10% |
| PBS (%) | 14% | 2% | 2% | 42% | 18% |
| **Regional (*n*)** | *257* | *259* | *232* | *174* | *98* |
| Mean excess cost per person | $4,281 | $1,630 | $22,605 | $15,490 | $64,324 |
| Hospital-based care (%) | 78% | 64% | 63% | 31% | 60% |
| MBS (%) | 13% | 13% | 22% | 15% | 12% |
| PBS (%) | 9% | 23% | 14% | 54% | 28% |
| **Distant metastases (*n*)** | *124* | *128* | *66* | *46* | *90* |
| Mean excess cost per person | $1,284 | $4,872 | $70,070 | $38,470 | $72,761 |
| Hospital-based care (%) | 5% | 63% | 44% | 26% | 55% |
| MBS (%) | 20% | 25% | 14% | 11% | 12% |
| PBS (%) | 75% | 12% | 41% | 63% | 33% |
| **Unknown stage (*n*)** | *153* | *153* | *141* | *116* | *42* |
| Mean excess cost per person | $933^a^ | -$1,227^a^ | $12,845 | $4,220 | $49,698 |
| Hospital-based care (%) | - | - | 47% | 34% | 68% |
| MBS (%) | - | - | 23% | 35% | 14% |
| PBS (%) | - | - | 30% | 32% | 18% |
| **All invasive cases (*n*)** | *3515* | *3551* | *3271* | *2870* | *655* |
| Mean excess cost per person | $1,002 | $1,174 | $8,858 | $3,566 | $53,928 |
| Hospital-based care (%) | 57% | 62% | 57% | 24% | 66% |
| MBS (%) | 29% | 32% | 29% | 27% | 11% |
| PBS (%) | 14% | 5% | 14% | 50% | 23% |

(continued)

| **Stage at diagnosis /**  Cost / Source | **1 year post-diagnosis** | **>1-2 years post-diagnosis** | **>2-3 years post-diagnosis** | **>3-4 years post-diagnosis** | **>4-5 years post-diagnosis** |
| --- | --- | --- | --- | --- | --- |
| ***In situ* (*n*)** | *4740* | *4155* | *3582* | *2994* | *2490* |
| Mean excess cost per person | $3,261 | $1,578 | $1,673 | $2,110 | $2,094 |
| Hospital-based care (%) | 50% | 55% | 51% | 61% | 59% |
| MBS (%) | 47% | 36% | 34% | 28% | 30% |
| PBS (%) | 3% | 8% | 15% | 11% | 11% |
| **Localised (*n*)** | *2877* | *2598* | *2302* | *2010* | *1694* |
| Mean excess cost per person | $6,335 | $2,724 | $3,646 | $3,496 | $2,640 |
| Hospital-based care (%) | 63% | 54% | 60% | 47% | 40% |
| MBS (%) | 34% | 31% | 23% | 24% | 25% |
| PBS (%) | 3% | 15% | 17% | 29% | 36% |
| **Regional (*n*)** | *255* | *211* | *162* | *137* | *107* |
| Mean excess cost per person | $29,344 | $18,929 | $16,770 | $17,718 | $13,652 |
| Hospital-based care (%) | 65% | 44% | 37% | 33% | 38% |
| MBS (%) | 20% | 15% | 14% | 15% | 17% |
| PBS (%) | 15% | 40% | 49% | 52% | 45% |
| **Distant metastases (*n*)** | *128* | *59* | *43* | *34* | *23* |
| Mean excess cost per person | $68,183 | $59,433 | $22,396 | $36,491 | $33,235 |
| Hospital-based care (%) | 49% | 31% | 32% | 50% | 32% |
| MBS (%) | 13% | 9% | 21% | 13% | 9% |
| PBS (%) | 38% | 59% | 47% | 37% | 59% |
| **Unknown stage (*n*)** | *148* | *129* | *101* | *77* | *54* |
| Mean excess cost per person | $14,744 | $9,398 | $8,142 | $11,525 | $5,639 |
| Hospital-based care (%) | 47% | 49% | 59% | 71% | 59% |
| MBS (%) | 24% | 21% | 20% | 23% | 29% |
| PBS (%) | 29% | 30% | 21% | 6% | 13% |
| **All invasive cases (*n*)** | *3408* | *2997* | *2608* | *2258* | *1878* |
| Mean excess cost per person | $11,471 | $5,681 | $5,141 | $5,381 | $3,908 |
| Hospital-based care (%) | 59% | 45% | 52% | 46% | 39% |
| MBS (%) | 25% | 21% | 20% | 21% | 21% |
| PBS (%) | 17% | 34% | 27% | 33% | 40% |

*^a^ Mean excess costs for people with unknown stage were negative for some sources in the two years prior to diagnosis, so the percentages by source are not reported here.*

*MBS: Medicare Benefits Schedule; PBS: Pharmaceutical Benefits Scheme.*

*Hospital-based care includes admitted hospitalisations and emergency department presentations – the latter generally accounted for 2-3% of all excess costs, up to a maximum 5% for any stage and time period.*

*For the 10% of invasive melanoma cases who had a subsequent in situ melanoma, mean costs based on their first melanoma (invasive) diagnosis were $8907 in the initial phase, $2610 per year in the continuing phase, and $55806 in the terminal phase. For the 5% of in situ cases who had a subsequent invasive melanoma, mean costs by phase based on their first melanoma (in situ) diagnosis were $4487, $5296 and $58167, respectively.*

**Table F**. Summary statistics for excess costs by stage, phase of care and time relative to diagnosis, for 45 and Up Study participants diagnosed with melanoma 2006-2019**.**

|  | ***In situ* - Excess costs** | | | | **Localised stage - Excess costs** | | | |
| --- | --- | --- | --- | --- | --- | --- | --- | --- |
|  | **No. of cases** | **Mean (SD)** | **Median** | **Quartiles 1 to 3** | **No. of cases** | **Mean (SD)** | **Median** | **Quartiles 1 to 3** |
| Phase of care |  |  |  |  |  |  |  |  |
| Initial | 4706 | $2,794 ($17,964) | $803 | -$2,842 to $5,686 | 2832 | $5,574 ($20,852) | $2,531 | -$1,461 to $8,711 |
| Continuing (per year) | 4091 | $1,222 ($16,302) | -$582 | -$4,367 to $4,057 | 2534 | $1,798 ($17,272) | -$376 | -$3,995 to $4,464 |
| Terminal | 427 | $40,914 ($53,320) | $26,702 | $5,836 to $60,373 | 425 | $46,674 ($59,796) | $33,474 | $5,320 to $65,859 |
| Annual costs around diagnosis |  |  |  |  |  |  |  |  |
| >1-2 years before diagnosis | 5001 | $759 ($16,229) | -$801 | -$4,025 to $2,274 | 2981 | $661 ($16,532) | -$905 | -$4,186 to $2,313 |
| >0-1 year before diagnosis | 5026 | $658 ($17,143) | -$939 | -$4,288 to $2,465 | 3011 | $1,066 ($16,963) | -$840 | -$4,303 to $2,524 |
| 0-1 year after diagnosis | 4740 | $3,261 ($19,161) | $864 | -$2,797 to $5,888 | 2877 | $6,335 ($22,772) | $2,714 | -$1,431 to $9,084 |
| >1-2 years after diagnosis | 4155 | $1,578 ($20,823) | -$753 | -$4,418 to $3,134 | 2598 | $2,724 ($21,179) | -$499 | -$3,846 to $3,894 |
| >2-3 years after diagnosis | 3582 | $1,673 ($20,774) | -$831 | -$4,759 to $3,292 | 2302 | $3,646 ($24,024) | -$407 | -$3,678 to $4,271 |
| >3-4 years after diagnosis | 2994 | $2,110 ($20,615) | -$771 | -$4,599 to $3,322 | 2010 | $3,496 ($25,910) | -$590 | -$4,371 to $3,974 |
| >4-5 years after diagnosis | 2490 | $2,094 ($20,965) | -$751 | -$4,285 to $3,934 | 1694 | $2,640 ($23,049) | -$685 | -$4,293 to $3,297 |
| By month relative to diagnosis |  |  |  |  |  |  |  |  |
| Month 6 before diagnosis | 5026 | $100 ($3,706) | -$55 | -$242 to $98 | 3011 | $82 ($3,969) | -$62 | -$247 to $108 |
| Month 5 before diagnosis | 5026 | $105 ($3,582) | -$60 | -$245 to $91 | 3011 | $141 ($3,289) | -$66 | -$255 to $106 |
| Month 4 before diagnosis | 5026 | $8 ($2,929) | -$56 | -$257 to $92 | 3011 | $82 ($3,304) | -$56 | -$252 to $100 |
| Month 3 before diagnosis | 5026 | $47 ($3,333) | -$60 | -$265 to $87 | 3011 | $68 ($3,342) | -$59 | -$265 to $105 |
| Month 2 before diagnosis | 5026 | -$1 ($2,842) | -$54 | -$230 to $91 | 3011 | $70 ($2,977) | -$48 | -$237 to $112 |
| Month 1 before diagnosis | 5026 | $13 ($2,970) | -$12 | -$207 to $198 | 3011 | $65 ($3,188) | $0 | -$193 to $229 |
| Month 1 after diagnosis | 5026 | $1,291 ($3,602) | $727 | $320 to $1,241 | 3011 | $2,465 ($4,226) | $1,068 | $540 to $3,937 |
| Month 2 after diagnosis | 5026 | $543 ($3,514) | $7 | -$180 to $420 | 3011 | $1,279 ($4,527) | $72 | -$143 to $1,110 |
| Month 3 after diagnosis | 5025 | $254 ($3,558) | -$39 | -$235 to $195 | 3008 | $338 ($3,374) | -$27 | -$229 to $249 |
| Month 4 after diagnosis | 5020 | $67 ($3,332) | -$45 | -$237 to $139 | 3003 | $84 ($3,129) | -$43 | -$242 to $141 |
| Month 5 after diagnosis | 5020 | $41 ($3,147) | -$54 | -$249 to $131 | 2999 | $229 ($3,562) | -$38 | -$230 to $179 |
| Month 6 after diagnosis | 5018 | $163 ($4,629) | -$48 | -$244 to $146 | 2992 | $191 ($3,453) | -$34 | -$232 to $174 |
| By month relative to death |  |  |  |  |  |  |  |  |
| Month 6 before death | 460 | $2,389 ($7,464) | $119 | -$264 to $1,840 | 437 | $2,606 ($8,536) | $117 | -$272 to $1,676 |
| Month 5 before death | 454 | $3,313 ($11,324) | $132 | -$171 to $1,685 | 432 | $3,649 ($10,076) | $215 | -$164 to $2,699 |
| Month 4 before death | 447 | $3,721 ($11,977) | $206 | -$176 to $2,430 | 430 | $3,309 ($10,800) | $250 | -$155 to $2,853 |
| Month 3 before death | 448 | $3,833 ($9,373) | $285 | -$107 to $3,867 | 430 | $5,207 ($14,151) | $248 | -$159 to $3,746 |
| Month 2 before death | 439 | $4,666 ($9,658) | $415 | -$53 to $6,999 | 429 | $5,842 ($11,236) | $751 | $31 to $8,080 |
| Final month of life | 429 | $14,205 ($19,400) | $8,945 | $644 to $21,319 | 425 | $16,520 ($23,882) | $10,148 | $607 to $23,989 |

|  | **Regional stage - Excess costs** | | | | **Distant metastases - Excess costs** | | | |
| --- | --- | --- | --- | --- | --- | --- | --- | --- |
|  | **No. of cases** | **Mean (SD)** | **Median** | **Quartiles 1 to 3** | **No. of cases** | **Mean (SD)** | **Median** | **Quartiles 1 to 3** |
| Phase of care |  |  |  |  |  |  |  |  |
| Initial | 232 | $22,605 ($35,546) | $10,894 | $5,014 to $28,560 | 66 | $70,070 ($74,971) | $39,733 | $20,486 to $97,577 |
| Continuing (per year) | 174 | $15,490 ($37,784) | $2,907 | -$1,191 to $17,047 | 46 | $38,470 ($55,890) | $22,229 | $1,486 to $53,020 |
| Terminal | 98 | $64,324 ($78,104) | $42,724 | $21,980 to $92,235 | 90 | $72,761 ($81,711) | $41,317 | $24,174 to $87,797 |
| Annual costs around diagnosis |  |  |  |  |  |  |  |  |
| >1-2 years before diagnosis | 257 | $4,281 ($22,938) | -$571 | -$3,745 to $3,873 | 124 | $1,284 ($15,732) | -$471 | -$4,561 to $3,706 |
| >0-1 year before diagnosis | 259 | $1,630 ($22,936) | -$1,588 | -$4,795 to $2,337 | 128 | $4,872 ($19,480) | $613 | -$2,170 to $7,259 |
| 0-1 year after diagnosis | 255 | $29,344 ($43,314) | $18,010 | $5,830 to $37,746 | 128 | $68,183 ($80,476) | $39,426 | $22,122 to $83,545 |
| >1-2 years after diagnosis | 211 | $18,929 ($51,273) | $3,196 | -$1,283 to $17,424 | 59 | $59,433 ($79,594) | $24,672 | $1,992 to $106,042 |
| >2-3 years after diagnosis | 162 | $16,770 ($48,748) | $2,094 | -$2,625 to $16,024 | 43 | $22,396 ($41,444) | $9,612 | $2,515 to $28,952 |
| >3-4 years after diagnosis | 137 | $17,718 ($56,367) | $1,528 | -$2,579 to $17,928 | 34 | $36,491 ($54,800) | $16,320 | $3,162 to $42,454 |
| >4-5 years after diagnosis | 107 | $13,652 ($46,539) | $556 | -$3,470 to $10,584 | 23 | $33,235 ($68,802) | $6,222 | -$739 to $34,297 |
| By month relative to diagnosis |  |  |  |  |  |  |  |  |
| Month 6 before diagnosis | 259 | -$155 ($2,153) | -$56 | -$278 to $137 | 128 | $267 ($5,275) | -$29 | -$302 to $173 |
| Month 5 before diagnosis | 259 | -$96 ($3,035) | -$55 | -$256 to $121 | 128 | $169 ($1,813) | $17 | -$184 to $187 |
| Month 4 before diagnosis | 259 | -$20 ($2,864) | -$85 | -$272 to $93 | 128 | $512 ($4,356) | -$46 | -$244 to $142 |
| Month 3 before diagnosis | 259 | $239 ($4,673) | -$57 | -$269 to $123 | 128 | $644 ($4,932) | -$22 | -$220 to $179 |
| Month 2 before diagnosis | 259 | $357 ($6,832) | -$40 | -$310 to $157 | 128 | $787 ($3,557) | $19 | -$218 to $406 |
| Month 1 before diagnosis | 259 | $148 ($3,399) | $15 | -$198 to $293 | 128 | $2,101 ($5,955) | $796 | -$45 to $2,212 |
| Month 1 after diagnosis | 259 | $6,420 ($8,298) | $4,183 | $867 to $8,317 | 128 | $14,703 ($19,804) | $7,997 | $2,495 to $18,774 |
| Month 2 after diagnosis | 259 | $5,000 ($8,824) | $1,977 | $38 to $7,119 | 123 | $10,485 ($17,900) | $4,304 | $388 to $15,781 |
| Month 3 after diagnosis | 259 | $2,928 ($6,895) | $261 | -$97 to $3,920 | 107 | $9,483 ($16,038) | $2,113 | $449 to $16,202 |
| Month 4 after diagnosis | 256 | $2,065 ($6,976) | $54 | -$157 to $1,121 | 100 | $7,371 ($13,373) | $566 | $29 to $10,518 |
| Month 5 after diagnosis | 255 | $1,998 ($8,165) | $40 | -$190 to $823 | 93 | $6,870 ($12,857) | $1,117 | $37 to $10,181 |
| Month 6 after diagnosis | 250 | $1,386 ($7,717) | $79 | -$148 to $579 | 87 | $4,516 ($9,551) | $1,008 | -$11 to $4,774 |
| By month relative to death |  |  |  |  |  |  |  |  |
| Month 6 before death | 93 | $4,868 ($16,921) | $504 | -$43 to $4,076 | 51 | $8,052 ($11,863) | $2,240 | $187 to $19,116 |
| Month 5 before death | 96 | $5,740 ($15,653) | $744 | -$25 to $6,369 | 57 | $7,435 ($13,022) | $1,855 | $232 to $10,374 |
| Month 4 before death | 97 | $5,162 ($11,119) | $1,004 | $62 to $5,534 | 64 | $7,580 ($13,326) | $2,618 | $642 to $7,816 |
| Month 3 before death | 99 | $7,915 ($14,865) | $2,424 | $68 to $10,665 | 70 | $9,412 ($16,471) | $2,468 | $823 to $12,374 |
| Month 2 before death | 98 | $8,883 ($16,835) | $2,445 | $174 to $10,128 | 85 | $13,147 ($18,666) | $7,637 | $1,962 to $16,731 |
| Final month of life | 98 | $15,434 ($17,245) | $10,439 | $1,221 to $26,439 | 90 | $19,299 ($22,884) | $14,082 | $1,782 to $28,124 |

|  | **Unknown stage - Excess costs** | | | | Table notes  SD: standard deviation  Costs reported in 2019 Australian dollars, for cases alive at the start of each time period.  Invasive melanoma cases with negative excess costs by phase of care: initial 31%, continuing 51%, terminal 13%.  In situ melanoma cases with negative excess costs by phase of care: initial 42%, continuing 55%, terminal 16%. |
| --- | --- | --- | --- | --- | --- |
|  | **No. of cases** | **Mean (SD)** | **Median** | **Quartiles 1 to 3** |  |
| Phase of care |  |  |  |  |  |
| Initial | 141 | $12,845 ($37,239) | $4,569 | -$1,676 to $15,088 |  |
| Continuing (per year) | 116 | $4,220 ($19,245) | $741 | -$2,978 to $10,547 |  |
| Terminal | 42 | $49,698 ($54,555) | $31,412 | $11,761 to $84,516 |  |
| Annual costs around diagnosis |  |  |  |  |  |
| >1-2 years before diagnosis | 153 | $933 ($15,717) | -$1,068 | -$5,078 to $2,465 |  |
| >0-1 year before diagnosis | 153 | -$1227 ($11,693) | -$1,245 | -$4,906 to $2,748 |  |
| 0-1 year after diagnosis | 148 | $14,744 ($38,050) | $4,782 | -$1,798 to $18,001 |  |
| >1-2 years after diagnosis | 129 | $9,398 ($29,128) | $736 | -$3,155 to $16,753 |  |
| >2-3 years after diagnosis | 101 | $8,142 ($32,847) | $232 | -$5,183 to $7,875 |  |
| >3-4 years after diagnosis | 77 | $11,525 ($33,672) | $2,028 | -$1,629 to $13,504 |  |
| >4-5 years after diagnosis | 54 | $5,639 ($26,037) | $11 | -$5,699 to $8,687 |  |
| By month relative to diagnosis |  |  |  |  |  |
| Month 6 before diagnosis | 153 | -$25 ($1,890) | -$88 | -$244 to $84 |  |
| Month 5 before diagnosis | 153 | -$318 ($1,760) | -$84 | -$269 to $85 |  |
| Month 4 before diagnosis | 153 | -$324 ($2,261) | -$100 | -$367 to $117 |  |
| Month 3 before diagnosis | 153 | -$344 ($1,660) | -$74 | -$248 to $190 |  |
| Month 2 before diagnosis | 153 | -$25 ($2,957) | -$80 | -$329 to $159 |  |
| Month 1 before diagnosis | 153 | $47 ($2,765) | $57 | -$230 to $366 |  |
| Month 1 after diagnosis | 153 | $2,475 ($4,987) | $1,034 | $294 to $3,499 |  |
| Month 2 after diagnosis | 153 | $1,330 ($4,688) | $127 | -$132 to $1,174 |  |
| Month 3 after diagnosis | 153 | $1,514 ($6,965) | $85 | -$136 to $844 |  |
| Month 4 after diagnosis | 152 | $648 ($3,437) | $4 | -$155 to $362 |  |
| Month 5 after diagnosis | 152 | $486 ($3,072) | -$4 | -$190 to $391 |  |
| Month 6 after diagnosis | 152 | $819 ($5,004) | -$12 | -$263 to $425 |  |
| By month relative to death |  |  |  |  |  |
| Month 6 before death | 45 | $3,261 ($6,480) | $307 | -$133 to $3,717 |  |
| Month 5 before death | 45 | $3,077 ($10,422) | $192 | -$241 to $3,195 |  |
| Month 4 before death | 45 | $4,475 ($10,132) | $688 | -$95 to $6,421 |  |
| Month 3 before death | 45 | $5,916 ($10,879) | $729 | -$52 to $9,050 |  |
| Month 2 before death | 43 | $5,351 ($8,356) | $1,321 | -$78 to $10,389 |  |
| Final month of life | 42 | $15,178 ($15,904) | $11,371 | $2,048 to $19,115 |  |

**Table G**. Mean excess costs by phase and years prior to diagnosis, by participant characteristics.

|  | **>1-2 years pre-diagnosis** | **1 year pre-diagnosis** | **Initial phase** | **Continuing phase (per year)** | **Terminal phase** | **n** |
| --- | --- | --- | --- | --- | --- | --- |
| **Invasive cases** | **$1,002** | **$1,174** | **$8,858** | **$3,566** | **$53,928** | 3551 |
| Age at diagnosis (years) |  |  |  |  |  |  |
| 45-59 | $310 | $486 | $6,450 | $1,968 | $125,240 | 652 |
| 60-69 | $645 | $1,465 | $8,766 | $3,114 | $88,326 | 1178 |
| 70-79 | $1,486 | $272 | $10,317 | $5,865 | $55,233 | 1035 |
| 80+ | $1,522 | $2,672 | $9,318 | $2,549 | $29,284 | 686 |
| Sex |  |  |  |  |  |  |
| Female | $580 | $767 | $8,583 | $1,931 | $49,939 | 1508 |
| Male | $1,313 | $1,473 | $9,065 | $4,796 | $55,657 | 2043 |
| Thickness |  |  |  |  |  |  |
| <0.8mm | $481 | $1,365 | $4,162 | $731 | $46,635 | 1861 |
| 0.8-1.0mm | -$385 | -$217 | $5,089 | $2,223 | $42,849 | 386 |
| >1-2mm | -$260 | -$257 | $10,655 | $3,656 | $48,066 | 509 |
| >2-4mm | $2,972 | $1,714 | $16,086 | $9,794 | $65,684 | 341 |
| >4mm | $4,903 | $1,440 | $19,089 | $11,673 | $50,078 | 247 |
| Unknown | $2,698 | $3,975 | $36,085 | $19,817 | $65,798 | 207 |
| Site |  |  |  |  |  |  |
| Trunk | $485 | $1,512 | $6,077 | $2,366 | $58,847 | 1179 |
| Upper limbs | $1,076 | $1,042 | $5,501 | $1,001 | $39,996 | 922 |
| Lower limbs | $881 | $1,004 | $10,585 | $2,654 | $51,644 | 682 |
| Face/neck/head | $1,994 | $511 | $11,288 | $7,676 | $50,138 | 652 |
| Other/unspecified | $706 | $3,224 | $53,154 | $35,124 | $73,483 | 116 |
| Histology |  |  |  |  |  |  |
| Superficial spreading | $418 | $692 | $6,528 | $1,146 | $53,159 | 1640 |
| Nodular | $3,075 | $1,700 | $16,597 | $10,990 | $65,966 | 398 |
| Lentigo | $888 | $1,264 | $4,375 | $2,873 | $43,310 | 434 |
| Other specified | $2,012 | $1,779 | $17,316 | $6,932 | $44,605 | 163 |
| No information | $975 | $1,627 | $10,442 | $4,900 | $52,156 | 916 |
| Charlson comorbidity score |  |  |  |  |  |  |
| 0 | -$384 | -$346 | $7,859 | $3,050 | $55,742 | 3170 |
| 1 | $10,917 | $9,034 | $20,262 | $8,068 | $63,848 | 206 |
| 2+ | $13,961 | $18,880 | $15,746 | $10,829 | $38,407 | 175 |
| Body Mass Index at baseline |  |  |  |  |  |  |
| Normal/Underweight (<25kg/m^2^) | -$302 | -$312 | $6,380 | $2,175 | $48,359 | 1141 |
| Overweight (25-<30kg/m^2^) | $557 | $1,232 | $8,532 | $4,183 | $53,403 | 1456 |
| Obese (≥30kg/m^2^) | $3,651 | $3,546 | $13,031 | $4,532 | $64,009 | 793 |
| Self-reported health (baseline) |  |  |  |  |  |  |
| Excellent | -$1,307 | -$1,323 | $6,711 | $1,978 | $64,688 | 559 |
| Very good | $197 | -$187 | $7,522 | $3,428 | $67,996 | 1386 |
| Good | $1,233 | $1,909 | $9,603 | $3,645 | $50,201 | 1130 |
| Fair/Poor | $7,031 | $7,538 | $15,799 | $7,560 | $44,132 | 358 |
| Smoking status (baseline) |  |  |  |  |  |  |
| Current | $3,099 | $2,056 | $13,629 | $5,480 | $65,160 | 164 |
| Former, quit <=15 years | $317 | $79 | $7,592 | $2,564 | $52,557 | 316 |
| Former, quit >15 years | $1,509 | $1,891 | $10,212 | $4,521 | $55,310 | 931 |
| Never | $718 | $953 | $8,098 | $3,172 | $52,244 | 2140 |
| Country of birth |  |  |  |  |  |  |
| Australia | $918 | $1,151 | $8,690 | $3,440 | $54,999 | 3051 |
| Other | $1,027 | $1,181 | $10,092 | $4,278 | $45,807 | 474 |
| Skin colour |  |  |  |  |  |  |
| Very fair | $1,798 | $383 | $10,134 | $4,804 | $62,779 | 754 |
| Fair | $603 | $1,471 | $8,213 | $2,906 | $49,992 | 2174 |
| Olive/Brown/Black | $1,525 | $967 | $9,721 | $3,911 | $57,391 | 582 |
| Tannability of skin |  |  |  |  |  |  |
| Very tannable skin | $371 | $1,074 | $8,682 | $2,565 | $57,878 | 641 |
| Moderately tannable | $1,017 | $946 | $8,397 | $3,721 | $52,346 | 1417 |
| Mildly tannable | $692 | $1,166 | $8,604 | $3,718 | $54,510 | 918 |
| Never tan | $2,209 | $1,676 | $10,870 | $4,190 | $55,594 | 510 |
| Remoteness of area of residence (baseline) |  |  |  |  |  |  |
| Major cities | $1,211 | $1,104 | $8,625 | $3,879 | $56,481 | 1789 |
| Inner regional | $520 | $1,000 | $9,343 | $3,306 | $50,108 | 1401 |
| Outer regional/Remote/Very remote | $1,831 | $2,194 | $8,131 | $3,028 | $55,013 | 361 |
| Socioeconomic level of area (baseline) |  |  |  |  |  |  |
| Quintile 1: Most disadvantaged | $2,113 | $2,701 | $10,170 | $5,149 | $51,222 | 705 |
| Quintile 2 | $1,083 | $76 | $6,853 | $2,445 | $49,244 | 728 |
| Quintile 3 | $615 | $2,195 | $9,141 | $3,669 | $62,177 | 651 |
| Quintile 4 | $130 | -$18 | $8,762 | $2,435 | $57,923 | 657 |
| Quintile 5: Least disadvantaged | $1,047 | $970 | $9,586 | $4,390 | $51,452 | 776 |
| Health insurance |  |  |  |  |  |  |
| Private | $1,157 | $1,244 | $9,776 | $4,307 | $57,203 | 2475 |
| Concession | $787 | $963 | $7,034 | $1,595 | $45,463 | 586 |
| None (public) | $475 | $1,053 | $5,435 | $1,346 | $59,011 | 423 |
| Highest education level attained |  |  |  |  |  |  |
| University+ | $615 | $1,228 | $8,151 | $3,788 | $58,115 | 808 |
| Certificate/Diploma/Trade/Apprentice | $1,416 | $452 | $8,687 | $4,001 | $53,870 | 1213 |
| High school certificate | -$282 | $807 | $9,797 | $3,630 | $49,139 | 306 |
| School certificate | $194 | $1,576 | $9,325 | $2,847 | $54,134 | 838 |
| No school certificate | $3,608 | $3,102 | $9,762 | $3,719 | $53,421 | 343 |
| ***In situ* cases** | **$759** | **$658** | **$2,794** | **$1,222** | **$40,914** | 5026 |
| Age at diagnosis (years) |  |  |  |  |  |  |
| 45-59 | $320 | $461 | $3,081 | $1,285 | $88,902 | 914 |
| 60-69 | $617 | $1,213 | $3,631 | $1,865 | $63,075 | 1770 |
| 70-79 | $853 | $71 | $2,169 | $1,451 | $43,124 | 1607 |
| 80+ | $1,440 | $848 | $1,723 | -$1,198 | $31,603 | 735 |
| Sex |  |  |  |  |  |  |
| Female | $735 | $807 | $2,056 | $1,004 | $43,385 | 2263 |
| Male | $778 | $536 | $3,395 | $1,401 | $39,759 | 2763 |
| Site (excluding n=13 other/unspecified) |  |  |  |  |  |  |
| Trunk | $1,092 | $679 | $2,473 | $1,748 | $41,424 | 1661 |
| Upper limbs | $769 | $607 | $2,295 | $975 | $40,891 | 1282 |
| Lower limbs | -$309 | $377 | $2,075 | -$144 | $44,725 | 627 |
| Face/neck/head | $842 | $837 | $3,887 | $1,320 | $39,220 | 1443 |
| Histology |  |  |  |  |  |  |
| Superficial spreading | $1,012 | $1,350 | $2,654 | $1,407 | $37,192 | 608 |
| Lentigo | $1,282 | $694 | $3,539 | $1,679 | $41,063 | 1888 |
| Other specified | $1,873 | $3,687 | $1,765 | -$1,055 | n.r. | 26 |
| Not specified | $294 | $431 | $2,263 | $821 | $40,718 | 2504 |
| Charlson comorbidity score |  |  |  |  |  |  |
| 0 | -$168 | -$359 | $2,137 | $527 | $40,011 | 4600 |
| 1 | $8,728 | $7,633 | $6,634 | $7,924 | $46,409 | 276 |
| 2+ | $14,492 | $19,010 | $17,923 | $14,391 | $39,449 | 150 |
| Body Mass Index at baseline |  |  |  |  |  |  |
| Normal/Underweight (<25kg/m^2^) | -$138 | -$343 | $1,523 | $276 | $35,703 | 1707 |
| Overweight (25-<30kg/m^2^) | $526 | $703 | $2,896 | $703 | $44,936 | 2099 |
| Obese (≥30kg/m^2^) | $2,446 | $2,778 | $5,567 | $4,210 | $40,732 | 990 |
| Self-reported health (baseline) |  |  |  |  |  |  |
| Excellent | -$1,300 | -$1,403 | $2,412 | -$764 | $37,819 | 870 |
| Very good | -$190 | -$478 | $1,011 | -$260 | $40,579 | 2101 |
| Good | $2,164 | $1,766 | $4,014 | $2,594 | $44,083 | 1497 |
| Fair/Poor | $3,823 | $6,055 | $7,975 | $7,376 | $39,004 | 423 |
| Smoking status (baseline) |  |  |  |  |  |  |
| Current | -$8 | -$810 | $1,282 | $1,224 | $44,870 | 196 |
| Former, quit <=15 years | $1,000 | $1,239 | $1,654 | $1,947 | $38,596 | 415 |
| Former, quit >15 years | $960 | $685 | $3,069 | $1,256 | $39,502 | 1304 |
| Never | $691 | $661 | $2,927 | $1,111 | $41,794 | 3111 |
| Country of birth |  |  |  |  |  |  |
| Australia | $801 | $659 | $2,802 | $1,048 | $41,100 | 4358 |
| Other | $565 | $634 | $2,859 | $2,200 | $42,796 | 635 |
| Skin colour |  |  |  |  |  |  |
| Very fair | $696 | $1,274 | $3,133 | $1,253 | $37,095 | 943 |
| Fair | $710 | $494 | $2,416 | $1,366 | $42,152 | 3086 |
| Olive/Brown/Black | $1,102 | $546 | $3,568 | $604 | $41,227 | 956 |
| Tannability of skin |  |  |  |  |  |  |
| Very tannable skin | $236 | $492 | $2,359 | $617 | $36,589 | 1028 |
| Moderately tannable | $1,064 | $363 | $3,116 | $1,156 | $41,550 | 2079 |
| Mildly tannable | $481 | $649 | $2,352 | $1,211 | $44,601 | 1251 |
| Never tan | $792 | $1,430 | $3,268 | $2,097 | $37,696 | 589 |
| Remoteness of area of residence (baseline) |  |  |  |  |  |  |
| Major cities | $1,267 | $591 | $3,474 | $1,553 | $44,739 | 2635 |
| Inner regional | $258 | $1,293 | $2,254 | $939 | $38,742 | 1914 |
| Outer regional/Remote/Very remote | -$31 | -$1,519 | $1,207 | $536 | $26,370 | 477 |
| Socioeconomic level of area (baseline) |  |  |  |  |  |  |
| Quintile 1: Most disadvantaged | $257 | $952 | $2,435 | $1,894 | $31,155 | 854 |
| Quintile 2 | $674 | $787 | $2,172 | $224 | $43,247 | 1007 |
| Quintile 3 | $930 | $242 | $3,297 | $1,578 | $36,175 | 954 |
| Quintile 4 | $1,127 | $860 | $2,152 | $691 | $46,805 | 902 |
| Quintile 5: Least disadvantaged | $878 | $538 | $3,750 | $1,663 | $50,399 | 1260 |
| Health insurance |  |  |  |  |  |  |
| Private | $1,057 | $904 | $3,348 | $1,699 | $46,892 | 3720 |
| Concession | $76 | $791 | $1,175 | $256 | $29,539 | 668 |
| None (public) | -$417 | -$789 | $1,178 | -$547 | $35,434 | 569 |
| Highest education level attained |  |  |  |  |  |  |
| University+ | $392 | $392 | $2,767 | $965 | $57,431 | 1234 |
| Certificate/Diploma/Trade/Apprentice | $445 | $938 | $3,252 | $1,456 | $40,014 | 1730 |
| High school certificate | $43 | $57 | $3,102 | $714 | $32,944 | 441 |
| School certificate | $960 | $703 | $2,087 | $461 | $37,453 | 1149 |
| No school certificate | $2,916 | $861 | $3,013 | $2,839 | $38,929 | 422 |

Costs for invasive cases are weighted by stage to match NSW stage distribution, numbers are otherwise unadjusted.

n.r.: not reported due to small cell sizes

**Table H**. Survival and cause of death information by stage, for 45 and Up Study participants diagnosed with invasive or *in situ* melanoma 2006-2019, and their matched controls**.**

|  |  | **Summary spread of disease at diagnosis** | | | | |
| --- | --- | --- | --- | --- | --- | --- |
|  | **Controls** | ***In situ*** | **Localised** | **Regional** | **Distant metastases** | **Unknown** |
| Number of people | 33868 | 5026 | 3011 | 259 | 128 | 153 |
| 1-year survival | 99% | 99% | 98% | 91% | 52% | 95% |
| 2-year survival | 97% | 98% | 93% | 70% | 36% | 81% |
| 5-year survival | 92% | 92% | 88% | 62% | 28% | 66% |
| Death record to Sep 2021, n (%) | 3734 (11%) | 560 (11%) | 525 (17%) | 112 (43%) | 93 (73%) | 51 (33%) |
| Cause of death available, n (%) | 2599 (8%) | 366 (7%) | 388 (13%) | 94 (36%) | 88 (69%) | 40 (26%) |
| *Melanoma / skin cancer, n (%^a^)* | *21 (1%)* | *13 (4%)* | *73 (19%)* | *52 (55%)* | *70 (80%)* | *16 (40%)* |
| *Other cancer, n (%^a^)* | *53 (2%)* | *95 (26%)* | *75 (19%)* | *12 (13%)* | *11 (13%)* | *8 (20%)* |
| *Heart disease, n (%^a^)* | *1135 (44%)* | *112 (31%)* | *109 (28%)* | *18 (19%)* | *n.r.* | *8 (20%)* |
| *Other causes, n (%^a^)* | *1390 (53%)* | *146 (40%)* | *131 (34%)* | *12 (13%)* | *n.r.* | *8 (20%)* |

*n.r.: not reported due to small cell sizes.*

*Note: Cause of death information from Cause of Death Unit Record File, data available to Dec 2019.*

*Survival estimates using Kaplan-Meier method, from diagnosis date. Survival time for controls starts from the diagnosis date of their matched case.*

*^a^ Primary cause of death, percentage listed is out of those with cause of death information available. Melanoma/skin cancer comprises ICD10 codes starting with C43 or C44, other cancer comprises all other codes starting with “C” or “D0”, heart disease comprises ICD10 codes starting with “I”.*

**Fig B.** Relative effects, p-values, and estimated excess costs from multivariable regression for excess costs for invasive melanoma cases diagnosed 2006-2019, for continuing and terminal phases.


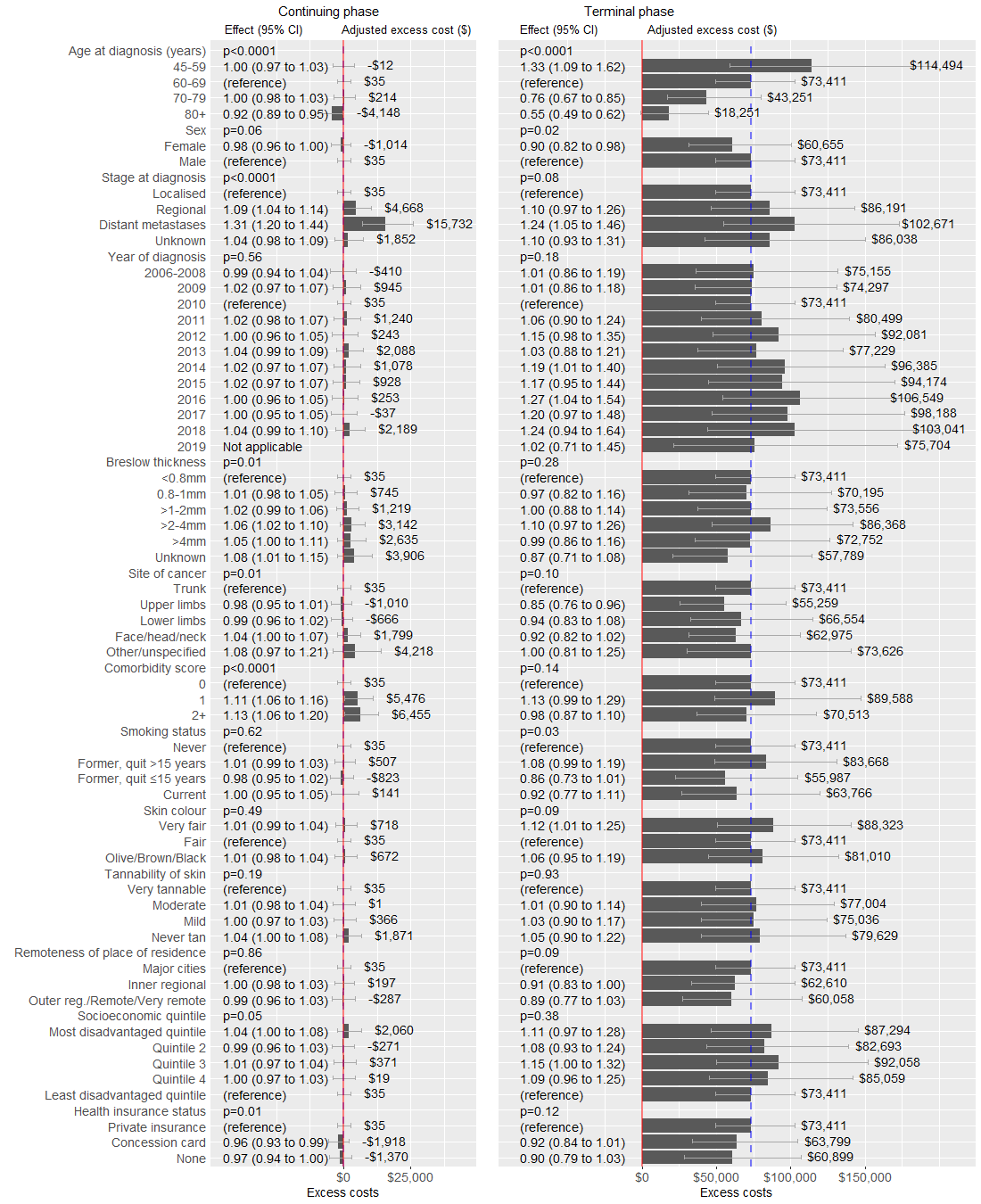


*Notes: The dashed vertical line is the estimated excess cost for a participant with invasive melanoma* ***who is in the reference category for all characteristics*** *(e.g. $73411 in the terminal phase for a male aged 60-69 with localised stage, etc.). For each category of a characteristic, the estimate shown reflects the adjusted excess cost for a case with all other characteristics in the reference category. To allow regression with non-negative values, models were constructed using an offset of +$50,000; this offset was then deducted to obtain the adjusted estimates shown in the figure.*

**Fig C.** Relative effects, p-values, and estimated excess costs from multivariable regression for excess costs for *in situ* melanoma cases diagnosed 2006-2019, for initial and continuing phases.


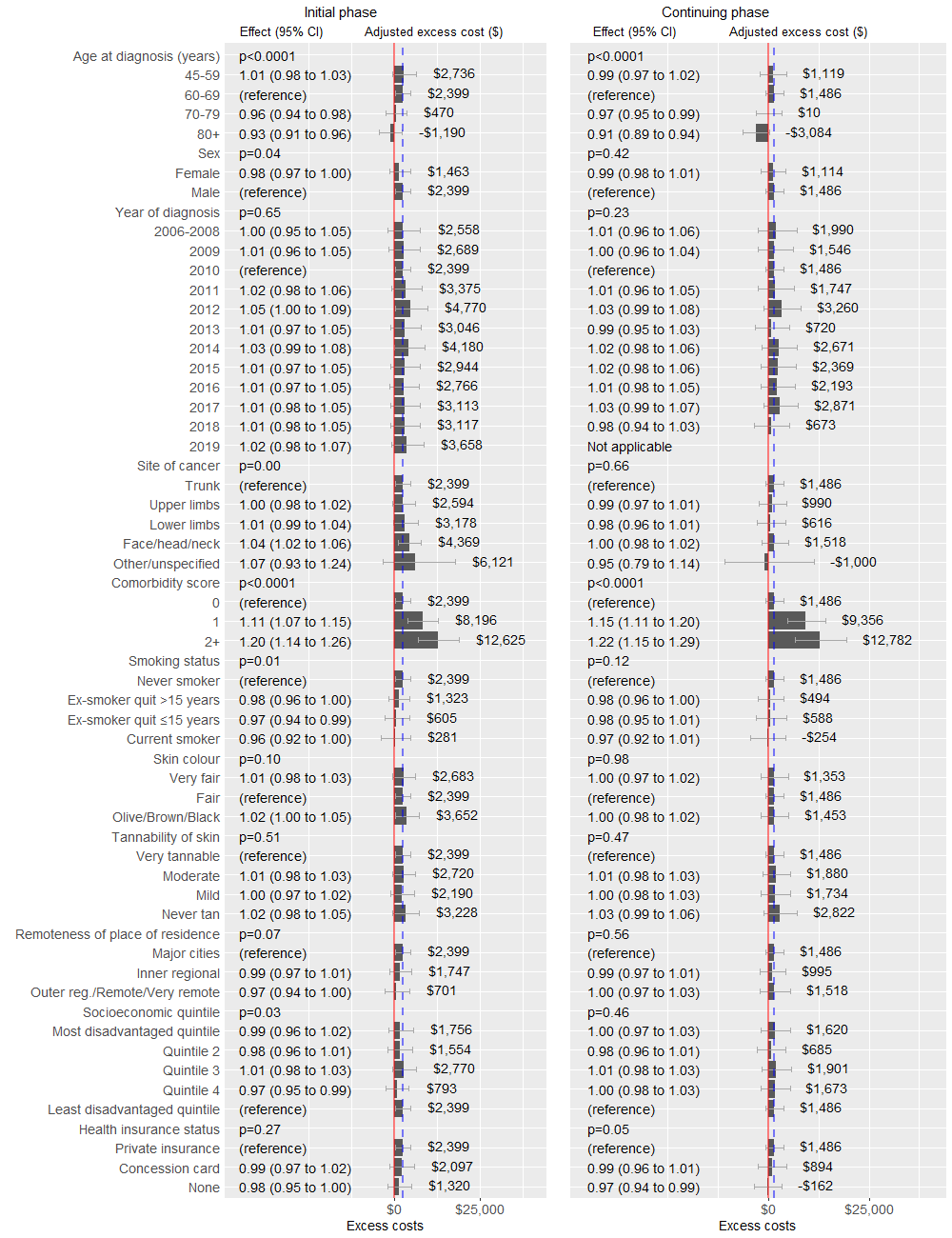


*Notes: The dashed vertical line is the estimated excess cost for a participant with in situ melanoma* ***who is in the reference category for all characteristics*** *(e.g. $2399 in the initial phase for a male aged 60-69 with no comorbidities, etc.). For each category of a characteristic, the estimate shown reflects the adjusted excess cost for a case with all other characteristics in the reference category. To allow regression with non-negative values, models were constructed using an offset of +$50,000; this offset was then deducted to obtain the adjusted estimates shown in the figure. Not reporting terminal phase for in situ due to small number of eligible cases.*

**Regression model sensitivity analyses**

We undertook a series of sensitivity analyses to test the robustness of the five main regression models (three phases for invasive, two phases for *in situ*). The final log-linked gamma regression models included an offset of the excess cost values by +$50,000 to allow for negative excess costs to be included in the calculation. In each phase >10% of cases had negative excess costs, and adding $50,000 meant there were negative “offset” costs for <0.5% of cases in each model, and this small proportion would be excluded due to the gamma regression requiring non-negative outcome values. In five separate sensitivity analyses, we tested the use of offsets of +$10,000 and +$100,000 for all excess costs, along with excluding records with negative excess costs, setting negative excess costs to $1, and using lognormal regression models.

Using an offset of +$100,000 gave very similar regression results to the reported main results.

Using an offset of +$10,000 resulted in the exclusion of 5%-11% of cases (who still had negative excess costs after the offset was added), which meant the lowest 5%-11% of excess cost observations were excluded but all of the highest costs remained. This skewed the input distribution and also meant there was a loss of information at the low end of costs. Additionally, this offset resulted in some highly influential values that ended up with an offset cost just above $0, and these needed to be excluded to keep the models stable. The association results themselves were generally similar in direction to the reported main results, but there were some more extreme effect sizes, and the occasional characteristic became highly statistically significant (or dropped well out of statistical significance) due to the exclusion of ~10% of cases that had particularly low costs.

There was an even greater loss of eligible cases and model information when excluding those with negative excess costs, taking out 13%-55% of cases in the different regression models. Again, the association results were similar in direction to the reported main results, but with some differences in effect size estimates and associations for a small number of variables moving in or out of statistical significance.

Setting negative excess costs to $1 also led to a loss of information from the data, with the distribution of negative excess costs being concentrated into one point, resulting in similar nullifying of effects at the low end of costs. This also substantially increased some of the mean costs, for example, the mean for localised cases in the initial phase increased by 45% from $5574 to $8105.

We also tested the use of lognormal regression, which had a similar result to the reported models, although the raw data appeared to fit the gamma distribution slightly better and the model statistics (Akaike Information Criterion, Bayesian Information Criterion) were generally better for the gamma regression models. Overall, the use of a log-linked gamma regression with an offset of +$50,000 tended to maximise the information that can be gleaned from the included cases. The other regression model formulations generally gave similar trends but with varying effect levels and random variations in significant characteristics.

As a sensitivity analysis, regression models were also tested for the inclusion of several other characteristics that, for brevity, are not in the reported figures. We tested the separate inclusion of histology, body mass index, self-reported health, country of birth, family history of melanoma, education, marital status, and more detailed thickness categories for <0.8mm. Some of these factors were associated with excess costs in different phases of care, but their inclusion had very little impact on the effect estimates and cost estimates for stage at diagnosis, and at most marginal impacts on the other characteristics included in the reported models. The strongest associations were for self-reported health in the initial and continuing phases (higher costs associated with fair/poor self-reported health, p<0.0001 for invasive and *in situ*), histology in the continuing phase (nodular had higher costs than superficial spreading, p=0.001 for invasive), and body mass index in the initial phase (higher costs for obese vs normal BMI, p=0.01 for invasive). None of the tested factors were associated with costs in the terminal phase (all p>0.05).

For all models we also tested year of diagnosis as a continuous variable to test for cost trends over time, along with using a diagnosis up-to-/post-2013 binary variable to test for differences brought on by changes in available/subsidised treatments. These variables were associated with excess costs in the terminal phase for invasive cases (p=0.002), as noted in the main manuscript, but they made little impact on the overall cost estimates for each stage at diagnosis. We also tested the inclusion of cause of death information in the terminal phase for people with invasive melanoma who had cause of death information available (n=563), and found higher costs were associated with people dying from melanoma or skin cancer compared to other causes (p=0.002). This slightly attenuated the main association with age (still p<0.0001), other variables were largely unchanged, including the trend by year in a separate test (still p=0.002), although the coefficient for stage at diagnosis became clearly non-significant (was p=0.08, became p=0.46). Among people in the terminal phase with cause of death information, 35% of those who died from melanoma/skin cancer had a record of immunotherapy and/or targeted therapy for melanoma, compared to 2% of people dying from other causes.

**Table I**. Total excess cost estimates in 2023 for all people in Australia diagnosed with melanoma during 2019-2023.

|  | **Year of diagnosis** | | | | |  |
| --- | --- | --- | --- | --- | --- | --- |
| **Stage at diagnosis** | **2023** | **2022** | **2021** | **2020** | **2019** | **Total** |
| Localised | $94,714,740 | $38,692,086 | $48,973,107 | $39,228,177 | $30,640,833 | $252,248,944 |
| Regional | $43,788,285 | $24,844,695 | $17,901,747 | $14,993,967 | $11,542,803 | $113,071,497 |
| Distant metastases | $48,995,934 | $21,276,994 | $6,056,956 | $7,551,389 | $6,173,124 | $90,054,397 |
| Unknown | $16,146,760 | $9,479,881 | $7,244,616 | $8,183,143 | $3,958,880 | $45,013,281 |
| **All invasive** | **$203,645,720** | **$94,293,656** | **$80,176,427** | **$69,956,676** | **$52,315,641** | **$500,388,119** |
| *In situ* | $99,175,040 | $45,385,647 | $45,272,861 | $52,454,608 | $47,269,688 | $289,557,845 |
| **All melanoma** | **$302,820,760** | **$139,679,303** | **$125,449,287** | **$122,411,285** | **$99,585,329** | **$789,945,964** |

*Invasive melanoma estimates use the incidence of invasive melanomas in Australia 2019-2023* (2)*, the stage distribution from NSW* (3)*, and the stage-specific survival rates and yearly costs from our study. In situ melanoma estimates are based on estimated incidence to 2021* (4) *with 5% annual increase in incidence to 2023, and survival and cost estimates from our study. Setting the annual in situ increase in incidence to 0% reduced the in situ estimate to $90 million for those diagnosed in 2023 and $278 million overall for people diagnosed with in situ melanoma in 2019-2023.*

**Supporting Information References**

1. Quan H, Li B, Couris CM, Fushimi K, Graham P, Hider P, et al. Updating and Validating the Charlson Comorbidity Index and Score for Risk Adjustment in Hospital Discharge Abstracts Using Data From 6 Countries. Am J Epidemiol. 2011 Mar 15;173(6):676–82.

2. Australian Institute of Health and Welfare. Australian Institute of Health and Welfare. [cited 2024 Sep 16]. Cancer data in Australia, Cancer summary data visualisation. Available from: https://www.aihw.gov.au/reports/cancer/cancer-data-in-australia/contents/summary-dashboard

3. Cancer Institute NSW. Cancer incidence, mortality and relative survival [Internet]. [cited 2024 Sep 16]. Available from: https://www.cancer.nsw.gov.au/research-and-data/cancer-data-and-statistics/data-available-now/cancer-statistics-nsw/cancer-incidence-mortality-survival

4. Australian Institute of Health and Welfare. Australian Institute of Health and Welfare. 2021 [cited 2024 Sep 16]. Cancer in Australia 2021. Available from: https://www.aihw.gov.au/reports/cancer/cancer-in-australia-2021/summary
