# Supplementary material for: Health system costs and systemic treatment patterns by disease stage for 8577 people diagnosed with melanoma in New South Wales, Australia 2006–2019
Source: PLoS One. 2026 Jul 13;21(7):e0353408. doi: 10.1371/journal.pone.0353408 (PMC13362142; doi:10.1371/journal.pone.0353408)
Supplement: Attachment — Submitted filename: Comments PONE.docx [file pone.0353408.s002.docx]

**The article entitled“Health system costs and systemic treatment patterns by disease stage for 8577 people diagnosed with melanoma in Australia 2005-2019”[** **PONE-D-25-47065], a questionnaire based study of health system costs in melanoma patients in Australia reveals that health system costs increased greatly with advancing melanoma stage, with higher costs linked to uptake of new immunotherapies/targeted therapies. Cost savings could arise from initiatives that detect and successfully treat melanomas at earlier stages, or prevent melanomas entirely The study has no novelty in the overall finding even though the data has been collected from a large number of patients over a long period. It may be submit to a more suitable journal. However there are certain comments that can be considered for improving the manuscript quality.**

1. Many of the references used for the study are taken from certain websites, the authenticity of those sites for data collection for a scientific manuscript should be cross checked.
2. The figures in the manuscript are of low quality, which could be improved during future submissions.
