# Supplementary material for: Health system costs and systemic treatment patterns by disease stage for 8577 people diagnosed with melanoma in New South Wales, Australia 2006–2019
Source: PLoS One. 2026 Jul 13;21(7):e0353408. doi: 10.1371/journal.pone.0353408 (PMC13362142; doi:10.1371/journal.pone.0353408)
Supplement: Attachment — Submitted filename: Melanoma costs - responses 20260424.docx [file pone.0353408.s004.docx]

**Responses to Editor and Reviewers’ comments**

**Editor**

**Comment**: This paper is very interesting and well written. The reviewers have considered several aspects that could improve the manuscript. I would like to stress the issue of the catchment area, which is not Australia. Please, try to modify considering this limited extension. The study, well designed as a case-control study, is nested in a large study, which probably is not representative of the entire country. Finally, in your text, you suggest there is a turning point in 2013, with new protocols and changes in treatment costs, possibly relevant. However, in the text you never considered a possible trend in the costs by year or period of diagnosis. Could you explain why?

**Response:** Thank you for the opportunity to revise and re-submit our paper. Thanks also to the editor and reviewers for your consideration and thoughtful suggestions, which have helped improve our work. We have added details and clarified areas as suggested, with a point-by-point response to all comments and details of changes provided in this document.

Regarding the catchment area, we have updated the title to reflect this by specifying, “New South Wales, Australia”, and also included clarifications on the sampling as described in the first response to reviewer 4.

In terms of cost trends by year, we did in fact include this in our analysis and made brief mention in the main manuscript, with more detail in the Supporting information describing the various sensitivity analyses. We have added the following (underlined) to the Methods and Results sections to make this more clear.

Materials and methods (Statistical analyses, paragraph 4): “We also undertook sensitivity analyses excluding participants with costs among the highest and lowest 1% and 5%, to test for differences in the associations without potentially extreme values, along with setting year of diagnosis as a continuous variable to test for cost trends over time, and using a diagnosis up-to-/post-2013 binary variable to test for differences brought on by changes in available/subsidised treatments.”

Results (Costs, paragraph 3): “Higher costs in the terminal phase were mainly associated with younger age, with a trend of higher costs in more recent years. … There was no other association between year and costs by phase or invasive/*in situ* melanoma.”

Supporting information (after Figure C): “For all models we also tested year of diagnosis as a continuous variable to test for cost trends over time, along with using a diagnosis up-to-/post-2013 binary variable to test for differences brought on by changes in available/subsidised treatments. These variables were~~. This was only~~ associated with excess costs in the terminal phase for invasive cases (p=0.002), as noted in the main manuscript, but they ~~this also~~ made little impact on the overall cost ~~effect~~ estimates for each stage at diagnosis.”

**Reviewer #1**

**Comment:** This study quantifies healthcare costs and treatment patterns for cutaneous melanoma by stage, using a large Australian cohort from the most populated region. Melanoma is a major public health issue in Australia. Understanding factors driving the healthcare costs (individual and tumor characteristics, medical insurance, access to care, SES, etc) and increasing trends is very relevant. The findings highlight the substantial financial impact burden of advanced disease, particularly with the uptake of systemic and newer therapies. Findings from this work underscore the potential cost savings from early detection and prevention. Apart from its relevance, the background, methods, data obtained, as well as the supplementary material are all well prepared and clearly presented. The healthier state and higher education of the 45 and Up cohort compared to the general Australian population and other pitfalls or limitations are discussed. The conclusions are supported by the data obtained and could influence funding decisions and screening programs.

Justify and explain the use of gamma regression. Were assumptions met?

**Response:** Thank you for your positive feedback. The manuscript has been updated to explain the use of gamma regression and the testing of relevant assumptions as shown below, including a new section in the Supporting Information that explains the regression model sensitivity analyses.

Materials and methods (Statistical analyses, paragraph 4): “… using a multivariable gamma regression with a log link, simultaneously adjusting for all other covariates, and excluding participants with a missing value for any of the included covariates (~5% in each model). Gamma regression with a log link is a widely accepted method for modelling for health care costs, as it appropriately accounts for the right-skewed distribution of continuous cost values, including a long tail driven by a small proportion of patients with very high expenditure (28). … Due to potential~~ly~~ for negative excess costs for some people with cancer, in the regression analysis all cost estimates were “offset” by adding a set amount (+$50,000), so that all cost values were >$0 and could be included in the gamma regression which requires non-negative values. We undertook five separate sensitivity analyses to test the model structure, including an offset of +$10,000, an offset of +$100,000, excluding all negative excess costs, setting negative excess costs to $1, and regression models using lognormal regression (see Supporting Information).”

Supporting information (after Figure C): “Regression model sensitivity analyses: We undertook a series of sensitivity analyses to test the robustness of the five main regression models (three phases for invasive, two phases for *in situ*). The final log-linked gamma regression models included an offset of the excess cost values by +$50,000 to allow for negative excess costs to be included in the calculation. In ~~(in~~ each phase >10~~5~~% of cases had negative excess costs, and adding $50,000 meant there were negative “offset” costs for <0.5% of cases in each model, and this small proportion would be excluded due to the gamma regression requiring non-negative outcome values~~)~~. ~~Models excluded cases with missing values for any of the listed variables and any outlying costs (standardised Pearson residual <-4 or >5).~~ In five separate sensitivity analyses, we tested the use of offsets of +$10,000 and +$100,000 for all excess costs, along with excluding records with negative excess costs, setting negative excess costs to $1, and using lognormal regression models.

Using an offset of +$100,000 gave very similar regression results to the reported main results.

Using an offset of +$10,000 resulted in the exclusion of 5%-11% of cases (who still had negative excess costs after the offset was added), which meant the lowest 5%-11% of excess cost observations were excluded but all of the highest costs remained. This skewed the input distribution and also meant there was a loss of information at the low end of costs. Additionally, this offset resulted in some highly influential values that ended up with offset cost just above $0, and these needed to be excluded to keep the models stable. The association results themselves were generally similar in direction to the reported main results, but there were some more extreme effect sizes, and the occasional characteristic became highly statistically significant (or dropped well out of statistical significance) due to the exclusion of ~10% of cases that had particularly low costs.

There was an even greater loss of eligible cases and model information when excluding those with negative excess costs, taking out 13%-55% of cases in the different regression models. Again, the association results were similar in direction to the reported main results, but with some differences in effect size estimates and associations for a small number of variables moving in or out of statistical significance.

Setting negative excess costs to $1 also led to a loss of information from the data, with the distribution of negative excess costs being concentrated into one point, resulting in similar nullifying of effects at the low end of costs. This also substantially increased some of the mean costs, for example, the mean for localised cases in the initial phase increased by 45% from $5574 to $8105.

We also tested the use of lognormal regression, which had a similar result to the reported models, although the raw data appeared to fit the gamma distribution slightly better and the model statistics (Akaike Information Criterion, Bayesian Information Criterion) were generally better for the gamma regression models. Overall, the use of a log-linked gamma regression with an offset of +$50,000 tended to maximise the information that can be gleaned from the included cases. The other regression model formulations generally gave similar trends but with varying effect levels and random variations in significant characteristics.”

**Comment**: The authors found a mean excess costs of approximately $1K pp with melanoma in each of the two years before their diagnosis. Are the authors referring to screening due to the individuals phenotypic or genetic risk factors? Overall, this discussion paragraph can clarify it further. The mention of the potential contribution of over-diagnosed melanoma is appreciated.

**Response:** As described in the manuscript, the pre-diagnosis costs could be a result of higher rates of interacting with the health system among people who are subsequently diagnosed with melanoma. The excess costs method doesn’t look for specific healthcare items, it is a measure of overall additional healthcare use, so we aren’t specifically referring to screening (or any other specific healthcare items). We have added text to the discussion to clarify this point.

Discussion (paragraph 6): “Notably, we found mean excess costs of ~$1000 per person with melanoma in each of the two years before their diagnosis. The excess costs method does not identify specific healthcare items, such as screening or specialist visits; rather, it is a measure of the overall costs of additional healthcare use. Even allowing for some diagnostic costs, this finding suggests that in our cohort and potentially across Australia, people ~~being~~ diagnosed with melanoma might have a higher rate of interacting with the health system in general compared to those without melanoma.”

**Comment**: Please clarify / include the test referred to here: …’ There is … and an effective diagnostic test…”

**Response:** The manuscript has been updated to clarify the test and treatment.

Discussion (paragraph 7): “People already interacting with the health system for other reasons might be more likely to take part in skin cancer screening … . There is an effective treatment for early-stage disease (surgical excision) and an effective diagnostic test (visual skin examination with dermoscopy with histopathological review of an excision biopsy)”

**Comment**: Can the authors speculate on what unmeasured biases could exist in this work?

**Response:** The main areas of biases for which we could not fully control are likely to be the representativeness of the study cohort, in particular the inclusion of relatively healthy participants who are higher users of preventive health services. We have noted these in the discussion section, and clarified that some of the listed examples are among the unmeasured biases.

Discussion (paragraph 8): “This study has some limitations. The 45 and Up Study cohort is not directly representative of the whole Australian population, as study participants are generally healthier and have higher educational levels (42), and are potentially more frequent users of preventive health services. … Further, this is an observational study and is likely to have other unmeasured biases. For example, we~~We~~ were only able to analyse participants based on their first invasive melanoma diagnosis, and ~~we~~ did not have subsequent primary/recurrence/progression data, so some people might have higher costs due to additional cancers that we could not identify. Also, using the supplied summary stage groupings (local, regional, distant metastases at diagnosis) does not line up exactly with the more detailed clinical staging categories from the American Joint Committee on Cancer (44) that determine treatment recommendations, potentially obscuring a more detailed picture of costs by clinical stage, particularly for non-localised disease.”

**Comment**: First paragraph of the Discussion – consider adding whether the findings were obtained after adjusting for confounding factors such as comorbidities.?

**Response:** We have adjusted the text as shown below.

Discussion (paragraph 1): “In this large, population-based study of people diagnosed with melanoma in Australia, we found that health system costs were much higher for those with more advanced melanoma at diagnosis, both in the initial treatment phase and the continuing care phase, even after adjusting for confounding factors. Almost half of all study participants diagnosed with regional disease or ~~/~~ metastatic melanoma after 2013 had a record of immunotherapy and/or targeted therapy. The average costs for participants receiving these specific treatments were approximately $60,000 per year higher than costs for participants with a similar stage but not receiving these therapies, reflecting the potential for additional costs for the treatment itself and any ~~costs alongside the costs of~~ monitoring, investigations, tests, and adverse events relating to the treatment. These results emphasise the importance of prevention and early detection of melanoma, for both ~~the~~ health and economic benefits.”

**Reviewer #2**

**Comment:** The article entitled “Health system costs and systemic treatment patterns by disease stage for 8577 people diagnosed with melanoma in Australia 2005-2019”, a questionnaire based study of health system costs in melanoma patients in Australia reveals that health system costs increased greatly with advancing melanoma stage, with higher costs linked to uptake of new immunotherapies/targeted therapies. Cost savings could arise from initiatives that detect and successfully treat melanomas at earlier stages, or prevent melanomas entirely. The study has no novelty in the overall finding even though the data has been collected from a large number of patients over a long period. It may be submit to a more suitable journal.

**Response:** We disagree that there is no novelty in the overall findings of our paper. This is the first study in Australia to describe detailed costs of melanoma care with costs disaggregated by a range of informative and useful patient characteristics that are directly relevant to live, national health policy issues. The description of uptake of new therapies in the cohort is novel, as well as associations by participant characteristics. Furthermore, the scope of this journal explicitly includes research on the basis of scientific validity rather than perceived significance or novelty. These results will allow more accurate economic evaluations of melanoma care and skin cancer control in Australia, the country with the highest melanoma rates in the world. Indeed, as discussed in the manuscript, such evaluation work is planned as part of the government-funded national Roadmap for a potential melanoma screening program in Australia. As an example of the policy relevance and utility of this work, our parallel analysis on lung cancer costs published in 2020 (<https://doi.org/10.1371/journal.pone.0238018>) was integral to the health economic modelling that supported the recommendation for the National Lung Cancer Screening Program which commenced in 2025.

**Reviewer #3**

**Comment:** In the paper, Goldsbury et al performed a large, population-based analysis of health system costs and systemic treatment patterns for melanoma in Australia using linked administrative data from the 45 and Up Study. The findings demonstrate strong stage-based differences in costs and highlight the economic impact of immunotherapy and targeted therapies, consistent with prior literature. Several comments here.

1. The presence of non-trivial excess costs in the pre-diagnosis period is important and may bias post-diagnosis cost estimates, particularly for *in situ* and localised disease. The implications of this for interpretation of initial- and continuing-phase costs should be discussed more explicitly.

**Response:** We agree that this is an important consideration. As described in the manuscript, if the higher costs pre-diagnosis continue, this might inflate post-diagnosis cost levels. We have added text to the discussion to clarify this point and the stage- and phase-specific levels of potentially inflated estimates.

Discussion (paragraph 6): “Notably, we found mean excess costs of ~$1000 per person with melanoma in each of the two years before their diagnosis. The excess costs method does not identify specific healthcare items, such as screening or specialist visits; rather, it is a measure of the overall costs of additional healthcare use. Even allowing for some diagnostic costs, this finding suggests that in our cohort and potentially across Australia, people ~~being~~ diagnosed with melanoma might have a higher rate of interacting with the health system in general compared to those without melanoma. If that higher rate of health system interaction continues post-diagnosis, it ~~could~~ might also inflate the cost estimates for different treatment phases in this study (with ~~a~~ the potential inflation by stage and phase ~~of ~$1000 per year~~ accounting for approximately 30% ~~up to 50%~~ of initial phase costs and approximately 50% of continuing phase costs estimated for participants with *in situ* and localised disease, and approximately 10%-15% ~~but only up to 10%~~ of the initial and continuing phase costs estimated for participants with regional and distant disease at diagnosis). The increased rate of health system interaction could also contribute to potential overdiagnosis of melanomas.”

**Comment:** 2. The use of registry “summary stage” as a proxy for clinical stage should be acknowledged more clearly, particularly given evolving treatment practices for regional disease. This limitation may affect interpretation of treatment patterns and costs.

**Response:** We agree that data on AJCC clinical stage would be ideal for this analysis. However, summary stage at diagnosis is the only staging information made available by the state cancer registry, and NSW is one of the few cancer registries in Australia that makes any staging information available, thus the current study is still a substantial advance in cancer cost analyses in Australia. This aspect has been noted in the discussion section, and the use of summary stage as a proxy for clinical stage is noted in the methods section, we have adjusted the manuscript to more clearly acknowledge this information.

Materials and methods (Statistical analyses, first paragraph): “Where relevant, costs were analysed using weights so that the (proxy) stage distribution in our study matched the distribution of all invasive melanomas diagnosed in NSW during 2006-2020 (23).”

Materials and methods (Statistical analyses, third paragraph): “We used NSWCR to ascertain: summary spread of disease at diagnosis (a proxy for “stage”: *in situ*; localised; regional; distant metastases; unknown), …”

Discussion (paragraph 9): “Also, using the summary stage groupings does not line up exactly with the clinical staging categories that determine treatment recommendations, potentially obscuring the interpretation of the results, particularly for non-localised disease. However, the NSW Cancer Registry is one of the few cancer registries in Australia that routinely makes staging information available for analysis, so being able to differentiate between spread of disease at this less granular level for such a large cohort is still a substantial step forward for evaluation of melanoma care in Australia.”

**Comment:** 3. The handling of negative excess costs using a fixed offset for gamma regression requires clearer justification. Brief discussion of alternative modelling approaches or reassurance that conclusions are robust to modelling choice would strengthen confidence in the results.

**Response:** As fully described in the first response for Reviewer 1, we have made substantial additions to the manuscript to explain the use of gamma regression and the testing of relevant assumptions, including a new section in the Supporting Information outlining the sensitivity analyses using alternative modelling approaches.

**Comment:** 4. Comparisons between advanced-stage patients who did and did not receive immunotherapy/targeted therapy should be interpreted cautiously. Differences in survival, health status, or comorbidities may partly explain cost differences, and causal language should be avoided.

**Response:** We thank the reviewer for this suggestion and have adjusted the language to ensure there is no causal inference.

Abstract (Results): “Of 195 participants with regional/metastatic melanoma diagnosed 2014-2019, 80 (41%) had a record of immunotherapy/targeted therapy and their ~~mean~~ unadjusted per-person costs were approximately $60,000 higher annually than the 115 participants without these treatments.”

Discussion (paragraph 1): “The average costs for participants receiving these specific treatments were approximately $60,000 per year higher than costs for participants with similar stage not receiving these therapies, reflecting the potential for additional costs for the treatment itself and any ~~costs alongside the costs of~~ monitoring, investigations, tests, and adverse events relating to the treatment.”

**Reviewer #4**

**Comment:** Your paper, 'Health system costs and systemic treatment patterns by disease stage for 8,577 people diagnosed with melanoma in Australia, 2005–2019', makes a valuable contribution to the evaluation of healthcare costs for melanoma. The following comments are intended to improve its clarity and help you to achieve your aims more effectively.

Major comments. 1. The study population was sampled from Services Australia's Medicare enrolment database and represents ~11% of the population of New South Wales, Australia, aged 45 years and over. Therefore, it is not directly representative of the entire population. This limitation is highlighted in the discussion (452–455); however, in several sections of the paper, including the abstract (lines 89–92) and the discussion (lines 373–375, 498–500), you refer to the study cohort as population-based. The representativeness of the results should be clarified, as this affects their generalisability.

**Response:** Thank you for your positive feedback and thoughtful comments. We use the term “population-based” to describe the sampling frame of the 45 and Up Study that aimed to recruit a random population sample within the specified age range and state of residence. We have now clarified this in the manuscript as per the following.

Abstract: We describe the costs and selected treatments by stage of disease for people diagnosed with melanoma in a~~n~~ large Australian ~~population-based~~ cohort study.

Introduction (paragraph 4): “… using a distinct large-scale ~~population-based~~ cohort study with a population-based sampling frame …”

Discussion (second-last paragraph): “The study used a large longitudinal cohort with a population-based ~~cohort with~~ sampling frame and extensive information on participants …”

**Comment:** 2. The cost categories included in the analysis should be clarified, particularly with regard to:

a) the inclusion/exclusion of costs of services covered by private insurance;

b) the inclusion/exclusion of direct medical costs related to services provided in other settings, such as hospices, integrated home care and local residential care. In general, the issue of the completeness of the healthcare cost estimates provided by the study should be addressed in the discussion.

**Response:** The coverage of cost data is described in the discussion section, and we have added some notes as suggested. Costs covered by private health insurance are separate to health system costs, and are beyond the scope of this study.

Discussion (paragraph 10): “The costs we used for targeted therapy and immunotherapy are based on PBS listed prices, and could differ substantially from the actual costs to the government due to confidential pricing arrangements with pharmaceutical companies (44). We also have not included patients using ‘off label’ therapies that are not yet publicly funded, and therapies provided through participation in clinical trials. … We did not have healthcare information when people had interstate public hospital admissions, or records of care beyond that in admitted hospitalisations, public emergency departments or through MBS/PBS, such as community-based palliative care or residential aged care. Out-of-pocket healthcare costs incurred by individuals were outside the scope of this study.”

**Comment:** 3. The matching of cases and controls for the evaluation of excess costs is based on the following variables: date of birth (±5 years), sex (female/male), local government area of residence (153 areas in New South Wales) and smoking status. I would suggest matching by private insurance status as well. This variable seems to be available for both cases and controls given that: a) In the introduction, you explain that: 'The Australian health system includes a combination of a government-funded "universal" public system for permanent residents, supplemented by a private health system largely paid for by individuals, private health insurance premiums and government subsidies' (lines 70–72); b) in the Results section, you explain that participants with melanoma and control participants have different proportions of private insurance (lines 262–265); c) In the results section, you also found that costs are associated with having private health insurance (lines 311-313).

**Response:** This is an interesting suggestion and one that we deliberated over for some time prior to the analysis before deciding to not match on private health insurance.

We do have access to health insurance status *at the time of the baseline questionnaire*. However, we were mindful of two issues in deciding to not match on private health insurance *a prori*: adverse selection and overfitting. Of the two, adverse selection may be the most concerning. Australian private health insurance has several features that may be useful for matching but several others that are of concern. The prices for health insurance are ‘community rated’. This means that private health insurance providers are prohibited from using an individual’s characteristics, including their health status, to set premiums. The premiums are the same for the same bundle of insured products for all who wish to purchase it. This potentially gives rise to adverse selection, where those at high risk of health-related expenditure, are more likely to purchase health insurance.^1^ If this was widespread then matching on private health insurance would result in matching high-risk individuals with melanoma with individuals of high-risk of other pathologies and bias estimates downwards.

There are also taxation benefits in Australia from purchasing health insurance for some individuals, as they avoid an extra levy payable by those without private health insurance and this gives rise to the opposite bias – that those with private health insurance may be low risk, the so called ‘advantageous selection’. Different studies have found different relative impacts of those two biases (for example ^2,3^). The advantageous selection may overlap with some of our other matching criteria, such as location, and age, sex and smoking status - all correlated with the choice to have private health insurance.^3^

Thus, it is not clear whether including private health insurance in the matching process would reduce confounding or, by contrast, introduce a new source of confounding. For these reasons, we think that matching by health insurance status would lead to results that are difficult to interpret. We have included a brief description of this in the manuscript as per the following.

Discussion (paragraph 6): “Having private health insurance has been found to be associated with more specialist consults and hospital services in patients with melanoma (43). We considered matching by private health insurance, although given the complex relationship between health insurance and health service use it wasn’t clear if this would reduce confounding or introduce a new source of confounding.”

References

1. Afoakwah C, Byrnes J, Scuffham P, Nghiem S. Testing for selection bias and moral hazard in private health insurance: Evidence from a mixed public-private health system. *Health Economics* 2023, 32: 3–24. <https://doi.org/10.1002/hec.4605>

2. Nguyen L, Worthington A. Adverse selection in private health insurance. *Consumer Interests Annual* 2017; 63: 25–34.

3. Buchmueller TC, Fiebig DG, Jones G, Savage E. Preference heterogeneity and selection in private health insurance: The case of Australia. *Journal of Health Economics* 2013; 32: 757–767. <https://doi.org/10.1016/j.jhealeco.2013.05.001>

**Comment:** 4. You did not use the information about the cause of death to distinguish between costs in the terminal phase due to end-of-life treatments for melanoma and costs related to other diseases. If this information cannot be used for this purpose, and patients in the terminal phase (dying from melanoma) cannot be distinguished from censored cases (dying from other causes), then this limitation and its consequences in terms of biased cost estimation for the terminal phase of care should be addressed in the discussion.

**Response:** Thank you for this suggestion. We included another sensitivity analysis including cause of death (melanoma/skin cancer or other cause) in the terminal phase regression model for people who died up to December 2019, where cause of death information was available. We found an association between higher costs and dying from melanoma/skin cancer. We also found greater use of immunotherapies and targeted therapies for this group. This has been included in the manuscript as follows.

Materials and methods (Statistical analysis, paragraph 4): “We also tested the inclusion of cause of death (melanoma/skin cancer or other cause) in the regression model for the terminal phase, based on the subset of people dying to December 2019 who had cause of death information available.”

Results (Costs, paragraph 3): “Higher costs in the terminal phase were mainly associated with younger age, with a trend of higher costs in more recent years; for those with cause of death information, there were higher costs for people who died from melanoma or skin cancer compared to other causes (see Supporting Information).”

Discussion (paragraph 4): “… the terminal phase costs for participants with *in situ* or localised melanoma largely represents end-of-life healthcare costs for non-melanoma causes of death. For participants with cause of death information available, terminal phase costs were higher for those who died from melanoma/skin cancer, potentially due to greater use of more expensive treatments such as immunotherapy and targeted therapy towards the end of life.”

Supporting Information (Regression model sensitivity analyses, paragraph 6): “We also tested the inclusion of cause of death information in the terminal phase for people with invasive melanoma who had cause of death information available (n=563), and found higher costs were associated with people dying from melanoma or skin cancer compared to other causes (p=0.002). This slightly attenuated the main association with age (still p<0.0001), other variables were largely unchanged, including the trend by year in a separate test (still p=0.002), although the coefficient for stage at diagnosis became clearly non-significant (was p=0.08, became p=0.46). Among people in the terminal phase with cause of death information, 35% of those who died from melanoma/skin cancer had a record of immunotherapy and/or targeted therapy for melanoma, compared to 2% of people dying from other causes.”

**Comment:** 5. The materials and methods section does not make it clear if and how you dealt with patients with multiple primaries, particularly with regard to distinguishing costs related to melanoma cancer from costs due to multiple primaries.

**Response:** Unfortunately, there were no data available on multiple primary invasive melanomas, only the first primary invasive melanoma and any *in situ* diagnoses for an individual. So, we were unable to explore costs for multiple primaries. We have added some information on costs by phase for people who had both invasive and *in situ* melanoma.

Materials and methods (Study sample, paragraph 2): “Participants could have a single invasive melanoma recorded (data on diagnosis of multiple primary invasive melanomas~~ies~~ were not available), and/or multiple *in situ* melanomas, so participants with melanoma were classified according to their first melanoma diagnosis.”

Results (paragraph 2): “There were 3551 participants first diagnosed with invasive melanoma (10% of these had a subsequent diagnosis of *in situ* melanoma during the study period) … The other 5026 participants were first diagnosed with in situ melanoma (5% had a subsequent diagnosis of invasive melanoma during the study period) ….”

Supporting Information (Table E): “For the 10% of invasive melanoma cases who had a subsequent *in situ* melanoma, mean costs based on their first melanoma (invasive) diagnosis were $8907 in the initial phase, $2610 per year in the continuing phase, and $55806 in the terminal phase. For the 5% of *in situ* cases who had a subsequent invasive melanoma, mean costs by phase based on their first melanoma (*in situ*) diagnosis by phase were $4487, $5296 and $58167, respectively.”

**Comment:** Minor comments. 1. The study cohort includes people diagnosed with cancer between 2006 and 2019. This period is clearly identified in the Materials and Methods section, the Results section, and Figure 2. However, in other sections of the paper and in the title, the diagnosis period is given as 2005. This inconsistency should be addressed, and the period of diagnosis should be indicated consistently throughout the paper.

**Response:** The 45 and Up Study recruited people 2005-2009, but participants in 2005 were part of a pilot study and were excluded from analyses here. We have made the exclusion more clear, and adjusted the manuscript to show 2006 as the earliest year for cancer diagnoses, including in the title and Figure 1.

Materials and methods (Study sample, paragraph 1): “From the 45 and Up Study baseline cohort we excluded participants (Fig 2): who were in the 2005 pilot, who later withdrew, …”

**Comment:** 2. Acronyms should be fully defined the first time they are introduced. This is not the case for 'NSW', for example. This should be verified throughout the text.

**Response:** We have verified this throughout the manuscript.

**Comment:** 3. I suggest including a horizontal bar representing the overlapping period across data sources in Figure 1, identifying the time of diagnosis of cases in the study cohort.

**Response:** This has been included as shown below.

**Comment:** 4. I suggest illustrating:

a) the breakdown of phases of care and the different care patterns a patient might experience;

b) the time window used for cost evaluation in the different phases of care, using a figure rather than just text (lines 176–190).

**Response:** We have added the following figure and brief footnotes, and cited it in the manuscript.

*Fig 3. Hypothetical examples of follow-up time allocated to different phases of care, based on years since diagnosis and end of follow-up or death.*

*Initial phase: Up to 12 months from the date of diagnosis; Continuing phase: Time between initial phase and terminal phase or end of follow-up; Terminal phase: For people who died, the last 12 months of life. All are mutually exclusive.*

**Comment:** 5. The pharmaceutical target therapies from the PBS file are mentioned in the text using the labels of the active ingredient of the drug. It would be useful to also list the corresponding ATC codes in an appendix table, as this would help to reproduce the analysis.

**Response:** The ATC codes are included in the first appendix table, as noted in the manuscript.

Materials and Methods (Treatment): “A secondary aim was to describe melanoma-related treatment patterns, particularly the use of targeted therapies and immunotherapies, along with various other diagnostic and therapeutic procedures (Table A in S1 File).”
