# Supplementary material for: Health system costs and systemic treatment patterns by disease stage for 8577 people diagnosed with melanoma in New South Wales, Australia 2006–2019
Source: PLoS One. 2026 Jul 13;21(7):e0353408. doi: 10.1371/journal.pone.0353408 (PMC13362142; doi:10.1371/journal.pone.0353408)
Supplement: Peer review history

# Peer review: Decision Letter 0

**DOI:** 10.1371/journal.pone.0353408.r001

**Authors:** Eugenio Paci

16 Mar 2026

PONE-D-25-47065

Health system costs and systemic treatment patterns by disease stage for 8577 people diagnosed with melanoma in Australia 2005-2019

PLOS One

Dear Dr. Goldsbury,

Thank you for submitting your manuscript to PLOS ONE. After careful consideration, we feel that it has merit but does not fully meet PLOS ONE’s publication criteria as it currently stands. Therefore, we invite you to submit a revised version of the manuscript that addresses the points raised during the review process.

Please take a look at the editor comments below.

Please submit your revised manuscript by Apr 30 2026 11:59PM. If you will need more time than this to complete your revisions, please reply to this message or contact the journal office at plosone@plos.org. When you're ready to submit your revision, log on to https://www.editorialmanager.com/pone/ and select the 'Submissions Needing Revision' folder to locate your manuscript file.

Please include the following items when submitting your revised manuscript:

- A letter that responds to each point raised by the academic editor and reviewer(s). You should upload this letter as a separate file labeled 'Response to Reviewers'.
- A marked-up copy of your manuscript that highlights changes made to the original version. You should upload this as a separate file labeled 'Revised Manuscript with Track Changes'.
- An unmarked version of your revised paper without tracked changes. You should upload this as a separate file labeled 'Manuscript'.

If you would like to make changes to your financial disclosure, please include your updated statement in your cover letter. Guidelines for resubmitting your figure files are available below the reviewer comments at the end of this letter.

If applicable, we recommend that you deposit your laboratory protocols in protocols.io to enhance the reproducibility of your results. Protocols.io assigns your protocol its own identifier (DOI) so that it can be cited independently in the future. For instructions see: https://journals.plos.org/plosone/s/submission-guidelines#loc-laboratory-protocols. Additionally, PLOS ONE offers an option for publishing peer-reviewed Lab Protocol articles, which describe protocols hosted on protocols.io. Read more information on sharing protocols at https://plos.org/protocols?utm_medium=editorial-email&utm_source=authorletters&utm_campaign=protocols.

We look forward to receiving your revised manuscript.

Kind regards,

Eugenio Paci, MD

Academic Editor

PLOS One

Journal Requirements:

Journal Requirements:

When submitting your revision, we need you to address these additional requirements.

1. Please ensure that your manuscript meets PLOS ONE's style requirements, including those for file naming. The PLOS ONE style templates can be found at

https://journals.plos.org/plosone/s/file?id=wjVg/PLOSOne_formatting_sample_main_body.pdf and

https://journals.plos.org/plosone/s/file?id=ba62/PLOSOne_formatting_sample_title_authors_affiliations.pdf

2. If the reviewer comments include a recommendation to cite specific previously published works, please review and evaluate these publications to determine whether they are relevant and should be cited. There is no requirement to cite these works unless the editor has indicated otherwise.

Additional Editor Comments:

This paper is very interesting and well written. The reviewers have considered several aspects that could improve the manuscript. I would like to stress the issue of the catchment area, which is not Australia. Please, try to modify considering this limited extension. The study, well designed as a case-control study, is nested in a large study, which probably is not representative of the entire country. Finally, in your text, you suggest there is a turning point in 2013, with new protocols and changes in treatment costs, possibly relevant. However, in the text you never considered a possible trend in the costs by year or period of diagnosis. Could you explain why?

[Note: HTML markup is below. Please do not edit.]

Reviewers' comments:

Reviewer's Responses to Questions

**Comments to the Author**

1. Is the manuscript technically sound, and do the data support the conclusions?

The manuscript must describe a technically sound piece of scientific research with data that supports the conclusions. Experiments must have been conducted rigorously, with appropriate controls, replication, and sample sizes. The conclusions must be drawn appropriately based on the data presented.

Reviewer #1: Yes

Reviewer #2: Yes

Reviewer #3: Partly

Reviewer #4: Yes

**********

2. Has the statistical analysis been performed appropriately and rigorously?

Reviewer #1: Yes

Reviewer #2: I Don't Know

Reviewer #3: Yes

Reviewer #4: Yes

**********

3. Have the authors made all data underlying the findings in their manuscript fully available?

The PLOS Data policy requires authors to make all data underlying the findings described in their manuscript fully available without restriction, with rare exception (please refer to the Data Availability Statement in the manuscript PDF file). The data should be provided as part of the manuscript or its supporting information, or deposited to a public repository. For example, in addition to summary statistics, the data points behind means, medians and variance measures should be available. If there are restrictions on publicly sharing data—e.g. participant privacy or use of data from a third party—those must be specified.

Reviewer #1: Yes

Reviewer #2: Yes

Reviewer #3: Yes

Reviewer #4: No

**********

4. Is the manuscript presented in an intelligible fashion and written in standard English?

PLOS ONE does not copyedit accepted manuscripts, so the language in submitted articles must be clear, correct, and unambiguous. Any typographical or grammatical errors should be corrected at revision, so please note any specific errors here.

Reviewer #1: Yes

Reviewer #2: Yes

Reviewer #3: Yes

Reviewer #4: Yes

**********

5. Review Comments to the Author

Please use the space provided to explain your answers to the questions above. You may also include additional comments for the author, including concerns about dual publication, research ethics, or publication ethics. (Please upload your review as an attachment if it exceeds 20,000 characters)

Reviewer #1: PONE-D-25-47065: Health system costs and systemic treatment patterns by disease stage for 8577 people diagnosed with melanoma in Australia 2005-2019

Reviewer comments. This study quantifies healthcare costs and treatment patterns for cutaneous melanoma by stage, using a large Australian cohort from the most populated region. Melanoma is a major public health issue in Australia. Understanding factors driving the healthcare costs (individual and tumor characteristics, medical insurance, access to care, SES, etc) and increasing trends is very relevant. The findings highlight the substantial financial impact burden of advanced disease, particularly with the uptake of systemic and newer therapies. Findings from this work underscore the potential cost savings from early detection and prevention. Apart from its relevance, the background, methods, data obtained, as well as the supplementary material are all well prepared and clearly presented. The healthier state and higher education of the 45 and Up cohort compared to the general Australian population and other pitfalls or limitations are discussed. The conclusions are supported by the data obtained and could influence funding decisions and screening programs.

Specific comments:

- Justify and explain the use of gamma regression. Were assumptions met?

- The authors found a mean excess costs of approximately $1K pp with melanoma in each of the two years before their diagnosis. Are the authors referring to screening due to the individuals phenotypic or genetic risk factors? Overall, this discussion paragraph can clarify it further. The mention of the potential contribution of over-diagnosed melanoma is appreciated.

- Row 441: please clarify / include the test referred to here: …’ There is … and an effective diagnostic test…”

- Can the authors speculate on what unmeasured biases could exist in this work?

- First paragraph of the Discussion – consider adding whether the findings were obtained after adjusting for confounding factors such as comorbidities.

Reviewer #2: The article entitled“Health system costs and systemic treatment patterns by disease stage for 8577 people diagnosed with melanoma in Australia 2005-2019”[ PONE-D-25-47065], a questionnaire based study of health system costs in melanoma patients in Australia reveals that health system costs increased greatly with advancing melanoma stage, with higher costs linked to uptake of new immunotherapies/targeted therapies. Cost savings could arise from initiatives that detect and successfully treat melanomas at earlier stages, or prevent melanomas entirely The study has no novelty in the overall finding even though the data has been collected from a large number of patients over a long period. It may be submit to a more suitable journal.

Reviewer #3: In the paper, Goldsbury et al performed a large, population-based analysis of health system costs and systemic treatment patterns for melanoma in Australia using linked administrative data from the 45 and Up Study. The findings demonstrate strong stage-based differences in costs and highlight the economic impact of immunotherapy and targeted therapies, consistent with prior literature. Several comments here:

1. The presence of non-trivial excess costs in the pre-diagnosis period is important and may bias post-diagnosis cost estimates, particularly for in situ and localised disease. The implications of this for interpretation of initial- and continuing-phase costs should be discussed more explicitly.

2. The use of registry “summary stage” as a proxy for clinical stage should be acknowledged more clearly, particularly given evolving treatment practices for regional disease. This limitation may affect interpretation of treatment patterns and costs.

3. The handling of negative excess costs using a fixed offset for gamma regression requires clearer justification. Brief discussion of alternative modelling approaches or reassurance that conclusions are robust to modelling choice would strengthen confidence in the results.

4. Comparisons between advanced-stage patients who did and did not receive immunotherapy/targeted therapy should be interpreted cautiously. Differences in survival, health status, or comorbidities may partly explain cost differences, and causal language should be avoided.

Reviewer #4: Dear Author(s),

Your paper, 'Health system costs and systemic treatment patterns by disease stage for 8,577 people diagnosed with melanoma in Australia, 2005–2019', makes a valuable contribution to the evaluation of healthcare costs for melanoma.

The following comments are intended to improve its clarity and help you to achieve your aims more effectively.

Major comments:

1. The study population was sampled from Services Australia's Medicare enrolment database and represents ~11% of the population of New South Wales, Australia, aged 45 years and over. Therefore, it is not directly representative of the entire population. This limitation is highlighted in the discussion (452–455); however, in several sections of the paper, including the abstract (lines 89–92) and the discussion (lines 373–375, 498–500), you refer to the study cohort as population-based. The representativeness of the results should be clarified, as this affects their generalisability.

2. The cost categories included in the analysis should be clarified, particularly with regard to:

a) the inclusion/exclusion of costs of services covered by private insurance;

b) the inclusion/exclusion of direct medical costs related to services provided in other settings, such as hospices, integrated home care and local residential care. In general, the issue of the completeness of the healthcare cost estimates provided by the study should be addressed in the discussion.

3. The matching of cases and controls for the evaluation of excess costs is based on the following variables: date of birth (±5 years), sex (female/male), local government area of residence (153 areas in New South Wales) and smoking status. I would suggest matching by private insurance status as well. This variable seems to be available for both cases and controls given that:

a) In the introduction, you explain that: 'The Australian health system includes a combination of a government-funded "universal" public system for permanent residents, supplemented by a private health system largely paid for by individuals, private health insurance premiums and government subsidies' (lines 70–72);

b) in the Results section, you explain that participants with melanoma and control participants have different proportions of private insurance (lines 262–265);

c) In the results section, you also found that costs are associated with having private health insurance (lines 311–313).

4. You did not use the information about the cause of death to distinguish between costs in the terminal phase due to end-of-life treatments for melanoma and costs related to other diseases. If this information cannot be used for this purpose, and patients in the terminal phase (dying from melanoma) cannot be distinguished from censored cases (dying from other causes), then this limitation and its consequences in terms of biased cost estimation for the terminal phase of care should be addressed in the discussion.

5. The materials and methods section does not make it clear if and how you dealt with patients with multiple primaries, particularly with regard to distinguishing costs related to melanoma cancer from costs due to multiple primaries.

Minor comments:

1. The study cohort includes people diagnosed with cancer between 2006 and 2019. This period is clearly identified in the Materials and Methods section, the Results section, and Figure 2. However, in other sections of the paper and in the title, the diagnosis period is given as 2005. This inconsistency should be addressed, and the period of diagnosis should be indicated consistently throughout the paper.

2. Acronyms should be fully defined the first time they are introduced. This is not the case for 'NSW', for example. This should be verified throughout the text.

3. I suggest including a horizontal bar representing the overlapping period across data sources in Figure 1, identifying the time of diagnosis of cases in the study cohort.

4. I suggest illustrating:

a) the breakdown of phases of care and the different care patterns a patient might experience;

b) the time window used for cost evaluation in the different phases of care, using a figure rather than just text (lines 176–190).

5. The pharmaceutical target therapies from the PBS file are mentioned in the text using the labels of the active ingredient of the drug. It would be useful to also list the corresponding ATC codes in an appendix table, as this would help to reproduce the analysis.

**********

6. PLOS authors have the option to publish the peer review history of their article (what does this mean?). If published, this will include your full peer review and any attached files.

If you choose “no”, your identity will remain anonymous but your review may still be made public.

**Do you want your identity to be public for this peer review?**  For information about this choice, including consent withdrawal, please see our Privacy Policy.

Reviewer #1: No

Reviewer #2: No

Reviewer #3: No

Reviewer #4: **Yes:** Silvia Francisci

**********

[NOTE: If reviewer comments were submitted as an attachment file, they will be attached to this email and accessible via the submission site. Please log into your account, locate the manuscript record, and check for the action link "View Attachments". If this link does not appear, there are no attachment files.]

To ensure your figures meet our technical requirements, please review our figure guidelines: https://journals.plos.org/plosone/s/figures

You may also use PLOS’s free figure tool, NAAS, to help you prepare publication quality figures: https://journals.plos.org/plosone/s/figures#loc-tools-for-figure-preparation.

NAAS will assess whether your figures meet our technical requirements by comparing each figure against our figure specifications.

Attachment Submitted filename: Comments PONE.docx

# Author comment: Author response to Decision Letter 1

**DOI:** 10.1371/journal.pone.0353408.r002

13 May 2026

Please see the attached "Response to Reviewers" file.

Attachment Submitted filename: Melanoma costs - responses 20260424.docx

# Peer review: Decision Letter 1

**DOI:** 10.1371/journal.pone.0353408.r003

**Authors:** Eugenio Paci, Eugenio Paci

23 Jun 2026

Health system costs and systemic treatment patterns by disease stage for 8577 people diagnosed with melanoma in New South Wales, Australia 2006-2019

PONE-D-25-47065R1

Dear Dr. Goldsbury,

We’re pleased to inform you that your manuscript has been judged scientifically suitable for publication and will be formally accepted for publication once it meets all outstanding technical requirements.

Within one week, you’ll receive an e-mail detailing the required amendments. When these have been addressed, you’ll receive a formal acceptance letter and your manuscript will be scheduled for publication.

An invoice will be generated when your article is formally accepted. Please note, if your institution has a publishing partnership with PLOS and your article meets the relevant criteria, all or part of your publication costs will be covered. Please make sure your user information is up-to-date by logging into Editorial Manager at Editorial Manager® and clicking the ‘Update My Information' link at the top of the page. For questions related to billing, please contact billing support.

If your institution or institutions have a press office, please notify them about your upcoming paper to help maximize its impact. If they’ll be preparing press materials, please inform our press team as soon as possible -- no later than 48 hours after receiving the formal acceptance. Your manuscript will remain under strict press embargo until 2 pm Eastern Time on the date of publication. For more information, please contact onepress@plos.org.

Kind regards,

Eugenio Paci, MD

Academic Editor

PLOS One

Additional Editor Comments (optional):

This manuscript is a very interesting and important assessment of the excess cost attributable to advanced stage and the changes related to early diagnosis of melanoma. In particular, it is documented the impact on costs of new treatments. The authors stated the results "emphasise the importance of prevention and early detection of melanoma, for both health and economic benefits". There are other epidemiological aspects worth investigating, such as overdiagnosis and the reduction of advanced-stage disease attributable to screening, which both should be considered in future modeling. However, the demonstration by the authors of the excess cost in this large case series is, in my opinion, extremely helpful to improve our knowledge of melanoma early diagnosis.

Reviewers' comments:

Reviewer's Responses to Questions

**Comments to the Author**

1. If the authors have adequately addressed your comments raised in a previous round of review and you feel that this manuscript is now acceptable for publication, you may indicate that here to bypass the “Comments to the Author” section, enter your conflict of interest statement in the “Confidential to Editor” section, and submit your "Accept" recommendation.

Reviewer #1: All comments have been addressed

Reviewer #4: All comments have been addressed

**********

2. Is the manuscript technically sound, and do the data support the conclusions?

The manuscript must describe a technically sound piece of scientific research with data that supports the conclusions. Experiments must have been conducted rigorously, with appropriate controls, replication, and sample sizes. The conclusions must be drawn appropriately based on the data presented.

Reviewer #1: (No Response)

Reviewer #4: Yes

**********

3. Has the statistical analysis been performed appropriately and rigorously?

Reviewer #1: (No Response)

Reviewer #4: Yes

**********

4. Have the authors made all data underlying the findings in their manuscript fully available?

The PLOS Data policy requires authors to make all data underlying the findings described in their manuscript fully available without restriction, with rare exception (please refer to the Data Availability Statement in the manuscript PDF file). The data should be provided as part of the manuscript or its supporting information, or deposited to a public repository. For example, in addition to summary statistics, the data points behind means, medians and variance measures should be available. If there are restrictions on publicly sharing data—e.g. participant privacy or use of data from a third party—those must be specified.

Reviewer #1: (No Response)

Reviewer #4: Yes

**********

5. Is the manuscript presented in an intelligible fashion and written in standard English?

PLOS ONE does not copyedit accepted manuscripts, so the language in submitted articles must be clear, correct, and unambiguous. Any typographical or grammatical errors should be corrected at revision, so please note any specific errors here.

Reviewer #1: (No Response)

Reviewer #4: Yes

**********

6. Review Comments to the Author

Please use the space provided to explain your answers to the questions above. You may also include additional comments for the author, including concerns about dual publication, research ethics, or publication ethics. (Please upload your review as an attachment if it exceeds 20,000 characters)

Reviewer #1: (No Response)

Reviewer #4: all questions raised have been adequately addressed by the authors, the paper is clear and well written

**********

7. PLOS authors have the option to publish the peer review history of their article (what does this mean?). If published, this will include your full peer review and any attached files.

If you choose “no”, your identity will remain anonymous but your review may still be made public.

**Do you want your identity to be public for this peer review?**  For information about this choice, including consent withdrawal, please see our Privacy Policy.

Reviewer #1: No

Reviewer #4: **Yes:** Silvia Francisci

**********

# Editor report: Acceptance letter

**DOI:** 10.1371/journal.pone.0353408.r004

**Authors:** Eugenio Paci, Eugenio Paci

PONE-D-25-47065R1

PLOS One

Dear Dr. Goldsbury,

I'm pleased to inform you that your manuscript has been deemed suitable for publication in PLOS One. Congratulations! Your manuscript is now being handed over to our production team.

At this stage, our production department will prepare your paper for publication. This includes ensuring the following:

* All references, tables, and figures are properly cited

* All relevant supporting information is included in the manuscript submission,

* There are no issues that prevent the paper from being properly typeset

You will receive further instructions from the production team, including instructions on how to review your proof when it is ready. Please keep in mind that we are working through a large volume of accepted articles, so please give us a few days to review your paper and let you know the next and final steps.

Lastly, if your institution or institutions have a press office, please let them know about your upcoming paper now to help maximize its impact. If they'll be preparing press materials, please inform our press team within the next 48 hours. Your manuscript will remain under strict press embargo until 2 pm Eastern Time on the date of publication. For more information, please contact onepress@plos.org.

You will receive an invoice from PLOS for your publication fee after your manuscript has reached the completed accept phase. If you receive an email requesting payment before acceptance or for any other service, this may be a phishing scheme. Learn how to identify phishing emails and protect your accounts at https://explore.plos.org/phishing.

If we can help with anything else, please email us at customercare@plos.org.

Thank you for submitting your work to PLOS ONE and supporting open access.

Kind regards,

PLOS ONE Editorial Office Staff

on behalf of

Dr. Eugenio Paci

Academic Editor

PLOS One
